# Transcriptomic Analysis of Skin Color in Anole Lizards

**DOI:** 10.1093/gbe/evab110

**Published:** 2021-05-14

**Authors:** Pietro Longo Hollanda de Mello, Paul M Hime, Richard E Glor

**Affiliations:** 1 Department of Ecology and Evolutionary Biology, University of Kansas, Lawrence, KS, USA; 2 Biodiversity Institute and Natural History Museum, University of Kansas, Lawrence, KS, USA

**Keywords:** RNA-seq, *Anolis distichus*, differential expression, enrichment analyses, dewlap color, color pattern, SCARB1, CYP2J

## Abstract

Color and color pattern are critical for animal camouflage, reproduction, and defense. Few studies, however, have attempted to identify candidate genes for color and color pattern in squamate reptiles, a colorful group with over 10,000 species. We used comparative transcriptomic analyses between white, orange, and yellow skin in a color-polymorphic species of anole lizard to 1) identify candidate color and color-pattern genes in squamates and 2) assess if squamates share an underlying genetic basis for color and color pattern variation with other vertebrates. Squamates have three types of chromatophores that determine color pattern: guanine-filled iridophores, carotenoid- or pteridine-filled xanthophores/erythrophores, and melanin-filled melanophores. We identified 13 best candidate squamate color and color-pattern genes shared with other vertebrates: six genes linked to pigment synthesis pathways, and seven genes linked to chromatophore development and maintenance. In comparisons of expression profiles between pigment-rich and white skin, pigment-rich skin upregulated the pteridine pathway as well as xanthophore/erythrophore development and maintenance genes; in comparisons between orange and yellow skin, orange skin upregulated the pteridine and carotenoid pathways as well as melanophore maintenance genes. Our results corroborate the predictions that squamates can produce similar colors using distinct color-reflecting molecules, and that both color and color-pattern genes are likely conserved across vertebrates. Furthermore, this study provides a concise list of candidate genes for future functional verification, representing a first step in determining the genetic basis of color and color pattern in anoles.

Significance Statement

We know very little about the genetic basis for color and color pattern in squamate reptiles. Using transcriptomic comparisons between orange, yellow, and white skin from a color-polymorphic anole lizard, we identified 13 best candidate color and color-pattern genes that have been functionally verified in other vertebrates. In addition, we found an unexpected upregulation of the carotenoid pathway in orange skin relative to yellow skin. These results led us to propose two nonexclusive mechanisms that anoles might use to produce orange pigments. This study provides not only a list of candidate genes and pathways for future biochemical and functional assays but also supports for growing evidence that both color and color-pattern genes are conserved across vertebrates.

## Introduction

The vertebrate skin has two primary roles: to provide protection against the external environment and to allow organisms to regulate their osmotic balance (Alibardi 2003). In addition, the skin is home to pigment-containing and light-scattering cells known as chromatophores (Bagnara and Hadley 1973). The distribution of different types of chromatophores and their light-reflecting molecules produce the colors and color patterns that vertebrates rely on for crypsis, aposematism, or intraspecific communication (Leal and Fleishman 2004; Stuart-Fox et al. 2004). Although a vertebrate’s color and color pattern can be seen as a single phenotype, the genes responsible for the development, maintenance, and distribution of chromatophores throughout the skin (henceforth color-pattern genes) differ from the genes that chromatophores use to synthesize the light-reflecting molecules that produce vertebrate color (henceforth color genes).

Vertebrate color is determined by two nonexclusive mechanisms (Shawkey and Hill 2005): structural and pigmentary. Structural mechanisms produce color through the cohesive scattering of light by thin, symmetrically arranged nanoscale structures (e.g., Maia et al. 2009). Pigmentary mechanisms, on the other hand, produce colors through the selective absorption of light by one or more types of pigments (e.g., Steffen and McGraw 2007). These pigments are synthesized by genes from the broadly conserved melanin, carotenoid, and pteridine pathways, which when mutated produce many of the described differences in pigmentary color within and among closely related vertebrate species (e.g., Rosenblum et al. 2004; Braasch et al. 2007; Andrade et al. 2019; Gazda et al. 2020).

Vertebrate color pattern, meanwhile, is determined by the development, distribution, and maintenance of chromatophores throughout the skin (Patterson and Parichy 2019). A growing number of studies on the genetic basis of color pattern in model organisms have found that, like color genes, color-pattern genes also appear to be broadly conserved across vertebrates (Mills and Patterson 2009). Differently from color genes, however, mutations in color-pattern genes have been linked to highly deleterious pleiotropic effects in both mammals and fish (e.g., Hosoda et al. 1994). This is likely due to their neural crest cell origin, which also gives rise to cell lineages as diverse as craniofacial cartilage and bone cells, enteric neurons, among others (DuShane 1935). Furthermore, research in model organisms shows that the differentiation, migration, and maintenance of chromatophores results from a complex network of interacting pathways associated with multiple biological processes, rather than the relatively modular pathways that produce color-reflecting molecules within chromatophores (Irion et al. 2016; Patterson and Parichy 2019).

**Fig. 1.**
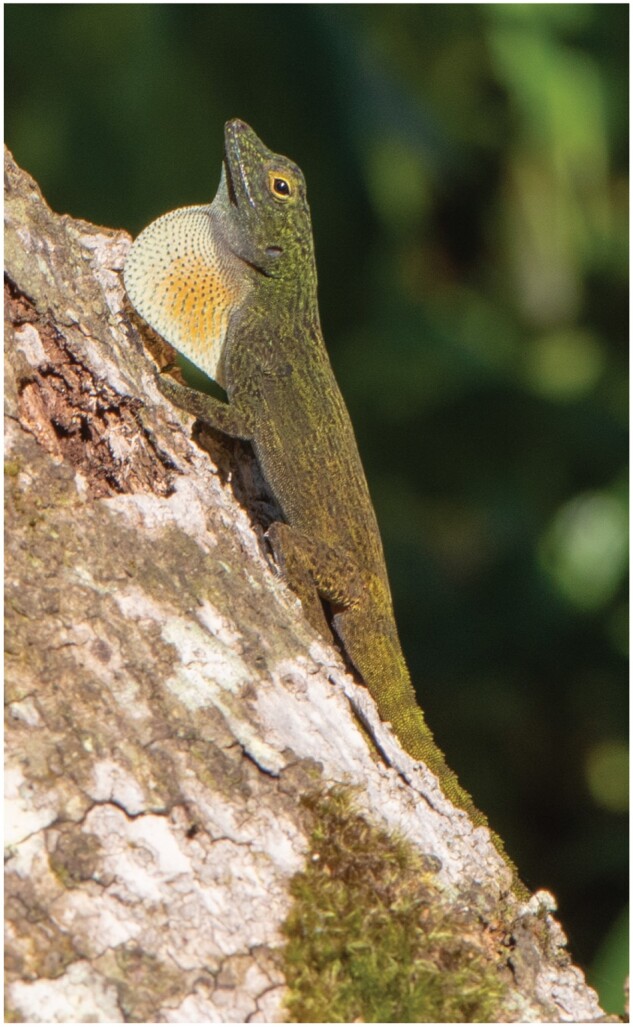
Glowing Ember Trunk Anole (*Anolis distichus favillarum*) with bicolored dewlap extended (Barahona, Peninsula, Dominican Republic). Notice the yellow margin surrounding the orange center that is typical of specimens from the contact zone between yellow and orange dewlapped populations. Photo by R.E.G.

To date, most studies on the genetics of vertebrate color and color pattern have focused on model organisms (e.g., zebrafish and mice), organisms that rely exclusively on a single type of chromatophore (i.e., melanophore), or organisms that deposit pigments in appendages like hairs and feathers (Hill and McGraw 2006; Hoekstra 2006; Patterson and Parichy 2019). Consequently, notwithstanding recent efforts (see below), no clear set of color genes or color-pattern genes have been established for squamates, a clade that includes over 10,000 species of lizards, snakes, and amphisbaenians (Olsson et al. 2013; Hasegawa et al. 2020). Squamates, like fish and amphibians, have three types of chromatophores: xanthophores/erythrophores, iridophores, and melanophores (DuShane 1935; Bagnara and Matsumoto 2006). The colors reflected by each of these chromatophores are determined by their pigments and structural elements. Yellow xanthophores and red erythrophores get their colors from pteridine-filled pterinosomes or carotenoid-filled lipid vesicles (Bagnara and Hadley 1973). Both of these pigments can reflect wavelengths in the yellow to red spectrum and both can be synthesized by a single chromatophore (Goodrich et al. 1941). Lizard melanophores, meanwhile, get their black tones from eumelanin-filled melanosomes (Seiji et al. 1961), while iridophores typically get their structural white colors from guanine platelet-filled organelles (Bagnara and Matsumoto 2006). The distribution of these three chromatophores throughout the skin produces the myriad color and color patterns we see across squamates (Bagnara et al. 1968; Alexander and Fahrenbach 1969), including the iconic color-shifting of chameleons, and the colorful extensible throatfans of anoles (fig. 1).

Given that the distribution of chromatophores and associated light-reflecting structures determines an organism’s color and color pattern, transcriptomic comparisons between differently colored patches of skin offer a promising avenue for identifying candidate color and color-pattern genes. At least two studies have used this approach to identify candidate color genes on Australian lizards (McLean et al. 2017, 2019). These studies produced largely nonoverlapping lists of candidate color genes, possibly because each species reflects similar colors with different pigment combinations: red skin in tawny lizards (*Ctenophorus decresii*, Agamidae) have relatively large amounts of drosopterins (a pteridine), while red skin in frill-neck lizards (*Chlamydosaurus kingii*, Agamidae) have both drosopterins and ketocarotenoids (ketolated carotenoids). In addition to these transcriptomic studies, a recent genomic study on the European common wall-lizard (*Podarcis muralis*, Lacertidae) identified a gene from the pteridine pathway [Sepiapterin Reductase (*SPR*)] as responsible for determining whether an animal has orange or yellow spots (Andrade et al. 2019). Studies on wild squamate populations like these, however, have generally not discussed a role for color-pattern genes, with recent efforts in identifying the genetic basis of squamate color-pattern coming from studies on captive-bred snake color-mutants (e.g., Ullate-Agote and Tzika 2021). Our primary aim, therefore, was to use transcriptomic analyses to identify both candidate color and color-pattern genes in a wild polymorphic population of anole lizards from Hispaniola.

Anoles are a species-rich group of Neotropical lizards that has long served as an important squamate model system (Losos 2009). Male anoles typically have colorful extensible throatfans, the dewlaps, that they extend during stereotypical behavioral displays (fig. 1) (Jenssen 1977). Most anoles have species-specific dewlap color and color pattern that are considered central to species recognition (Losos 2009). A few species, however, exhibit impressive dewlap color and color-pattern polymorphism (Underwood and Williams 1959; Leal and Fleishman 2004; Stapley et al. 2011; Ng, Landeen, et al. 2013; Prates et al. 2015). One such species is the Hispaniolan Trunk Anole (*Anolis distichus*), a widespread and common anole from Hispaniola and the Bahamas whose dewlaps can range from entirely pale yellow to dark red (fig. 1) (Schwartz 1968). Most dewlap variation in Hispaniolan trunk anoles is found among geographic populations that have been recognized as subspecies. Although some of these subspecies likely warrant status as full species, most appear to experience some degree of intergradation where they come into contact (Glor and Laport 2012; Geneva et al. 2015; MacGuigan et al. 2017; Myers et al. 2020). Moreover, a significant correlation between dewlap color and environmental variation across trunk anole populations suggests that dewlap color may be driven by selection for visibility across different environments rather than reflecting boundaries between reproductively isolated populations (Leal and Fleishman 2004; Ng et al. 2013).

We focus here on a subspecies of the Hispaniolan bark anole from the Barahona Peninsula in Southern Hispaniola: *Anolis distichus favillarum*, the “Glowing Ember Trunk Anole” (Schwartz 1968). This subspecies exhibits geographic dewlap color variation along an altitudinal and environmental gradient, while lacking any evidence for geographic genetic structure between populations (Glor and Laport 2012; Geneva et al. 2015; Ng et al. 2016). Orange dewlapped populations inhabit wetter upland environments, while yellow dewlapped populations inhabit drier coastal environments (Schwartz 1968). We recently identified several localities along this altitudinal transect that are home to individuals with intermediate phenotypes, which have dewlaps with orange centers and yellow margins (fig. 1). The combined effects of low genetic population structure and divergent selection for dewlap color across this wet-to-dry environmental transect makes the Glowing Ember Trunk Anole an ideal system to identify candidate color and color-pattern genes in squamates.

**Fig. 2.**
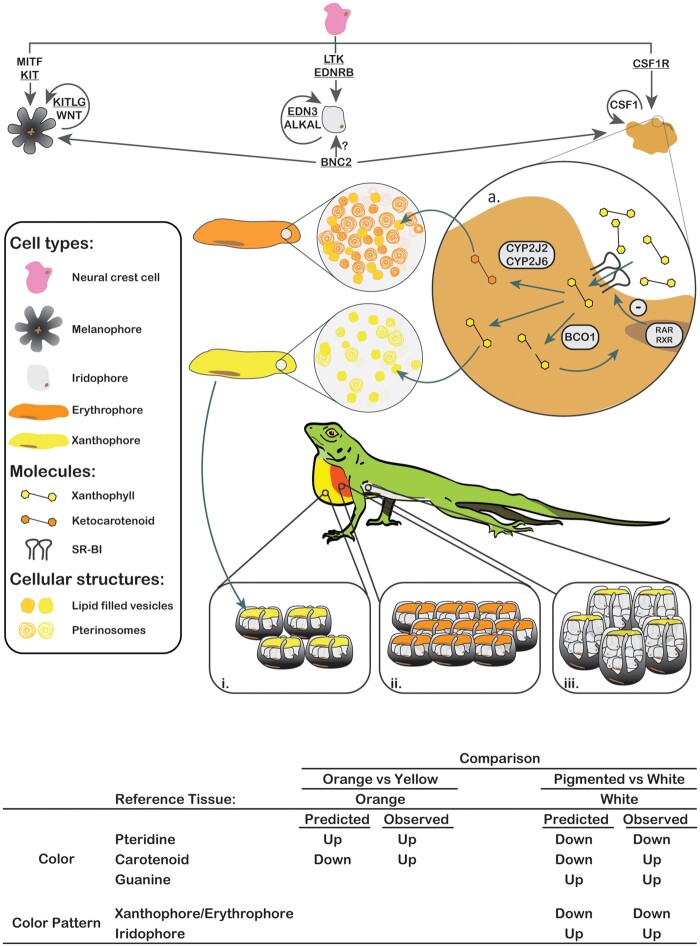
Schematic representation of the hypothesized genetic determinants for color and color pattern in the Glowing Ember Trunk Anole (*Anolis distichus favillarum*). All three types of dermal chromatophores originate through the migration and differentiation of neural crest cells. Arrows connecting the neural crest cells to each chromatophore represent genes responsible for chromatophore differentiation in zebrafish (reviewed in Patterson and Parichy 2019). Arrows connecting *BNC2* to chromatophores indicate that *BNC2* is produced by surrounding cells rather than chromatophores themselves. The genes we found to be differentially expressed are underlined. See table 1 for details. Inset “a.” depicts the hypothesized negative-feedback mechanism for carotenoid influx regulation we based on recent findings in mice (Widjaja-Adhi et al. 2015); this inset also illustrates the two candidate genes for ketolation we identified in *A. distichus*. Once dietary xanthophylls are scavenged from the blood by *SCARB1* they can either be broken down by *BCO1* and used in the synthesis of retinoic acid products (RAR/RXR), directly deposited in lipid vesicles, or, potentially, ketolated by either *CYP2J2* or *CYP2J6*. Our expression data suggests that erythrophores could hold both ketocarotenoids and drosopterins. Insets “i,” “ii,” and “iii” represent the expected composition of chromatophore units in yellow, orange, and white skin, respectively. Given our differential expression results, we expect orange skin to have more melanophores and erythrophores per unit area than yellow skin, and white skin to have more iridophores per unit area than pigmented skin. At the bottom of the figure we present a table with the predicted and observed gene expression patterns for each skin comparison.

To identify candidate genes and pathways associated with both color and color pattern in squamates, we combined differential expression tests and gene enrichment analyses in comparisons between white belly skin and orange and yellow dewlap skin from specimens of the Glowing Ember Trunk Anole (Dactyloidae; fig. 1). Given that white and pigment-rich skin in anoles are expected to differ both in chromatophore composition and in the reflecting structures they synthesize, we predicted that (fig. 2) i) differences in expression profiles between white and pigment-rich skin would be larger than those between orange and yellow skin. Furthermore, since chromatographic studies indicate that anoles typically use drosopterins to produce red colors, xanthophylls to produce yellow colors, and guanine platelets to produce white colors (Ortiz and Williams-Ashman 1963; Macedonia et al. 2000; Steffen and McGraw 2007; Alfonso et al. 2013), we predicted that in comparisons between white and pigment-rich skin: ii) white skin would upregulate the guanine synthesis pathway and iii) pigment-rich skin would upregulate the carotenoid (Provitamin-A) and pteridine pathways. Given that prior chromatographic studies in other anole species have suggested that orange and yellow colorations in anoles result from pteridines and carotenoids, respectively, we also predicted that in comparisons between orange and yellow skin: iv) orange skin would upregulate the pteridine pathway, while v) yellow skin would upregulate the carotenoid pathway. Lastly, based on the literature about color-pattern genes in zebrafish we predicted that in white vs. pigment-rich skin comparisons: vi) white skin would upregulate iridophores differentiation and maintenance genes, while vi) pigment-rich skin would upregulate xanthophores/erythrophores differentiation and maintenance genes.

Using a combination of enrichment analyses, differential expression tests, and a literature review we identified six best candidate color and seven best candidate color-pattern genes in squamates, which we highlight for future functional assessment. Among color genes were Scavenger Receptor Class B Member 1 (*SCARB1*), a sex-linked gene that encodes a lipid scavenger transmembrane protein previously linked to color in vertebrates, and two genes from the Cytochrome P450 2J family (*CYP2J*), which includes the gene responsible for the ketolation of carotenoids in bird liver and skin (Lopes et al. 2016; Mundy et al. 2016). As we expected, we found genes from the pteridine and carotenoid pathways to be differentially expressed across orange, yellow, and white skin colors. These genes, however, did not overlap with candidate color genes identified in previous studies in squamate coloration (McLean et al. 2017; Andrade et al. 2019; McLean et al. 2019). Color-pattern genes, on the other hand, were not only shared with findings in the distantly related zebrafish but also showed expression patterns consistent with those found in this model species (Patterson and Parichy, 2019). Our transcriptomic results, therefore, support the predictions that the genetic basis for color and color pattern are conserved across vertebrates, and that even though squamates can produce similar colors using distinct color-reflecting molecules, the expression patterns of genes responsible for the differentiation and maintenance of chromatophores appear to be conserved across vertebrates.

## Results and Discussion

### Skin with Different Colors Have Different Expression Profiles

**Fig. 3.**
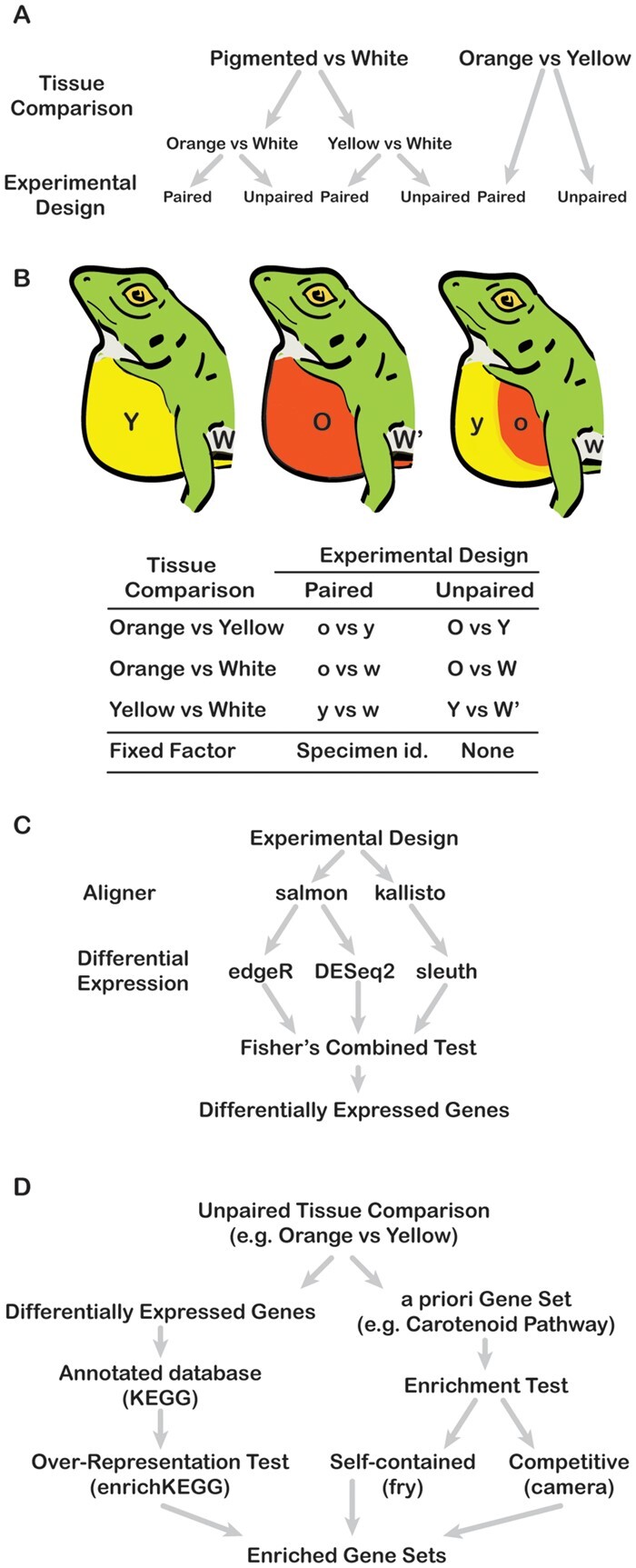
Experimental design layout for differential expression and genet set enrichment analyses. (A) The experimental designs we implemented for each skin comparison. (B) Illustration of yellow, orange, and bicolored dewlapped specimens, as well as the regions of the dewlap from which we sampled skin. Lower and capital letters represent skin used in paired and unpaired experimental designs, respectively. (C and D) The differential expression and gene set enrichment pipelines we implemented, respectively.

We performed differential expression analyses for three pairwise comparisons involving different skin colors: orange dewlap versus white belly, yellow dewlap versus white belly, and orange dewlap versus yellow dewlap (fig. 3*a* and *b*). All comparisons had three samples per skin color (supplementary tables S1 and S2, Supplementary material online). For each comparison, we ran three differential expression pipelines using both paired and unpaired experimental designs (fig. 3*c* and *d*; see Materials and Methods section). In the unpaired experimental designs, we collected samples of the two colors from different individuals; in the paired experimental design (as in paired *t*-tests), we collected both color samples from the same individual and used specimen identification as a fixed factor when fitting the generalized linear model (see Materials and Methods section). For each of the three paired color comparisons, we used Fisher’s combined test to identify which genes had expression profiles consistent with differential expression across all three pipelines and both types of experimental designs.

On average, we sequenced 27,306,758 raw reads per sample. After filtering and cleaning for contaminants, we kept a mean of 23,818,269 reads per sample, and aligned a mean of 77.5% (_**±**_ 5.5%) of these reads to the annotated transcriptome using salmon (Patro et al. 2017) (table S1, Supplementary material online). Of the 18,905 genes we annotated to the Glowing Ember Trunk Anole skin Transcriptome (Materials and Methods section), only 604 genes were differentially expressed across at least one of our three pairwise comparisons. The number of differentially expressed genes in a single pairwise comparison varied between 20 and 455. Moreover, principal component analyses (PCAs) of estimated read counts for all annotated genes (fig. S1, Supplementary material online) showed that most variance in estimated read counts (PC1) was associated with differences between specimens, while principal components that visually clustered samples based on color (PC2, 3, and 4) explained between 8.6 and 27.4% of the total variance.

In agreement with our prediction that differences in expression profiles would be larger between white and pigment-rich skin than between orange and yellow skin, we found more differentially expressed genes in comparisons between white and pigment-rich skin than between orange and yellow skin. Furthermore, PC axes that separated white from orange skin across paired and unpaired designs explained a larger proportion of the variance than the axes that visually separated white skin and yellow skin. These results suggest that orange and yellow skin expression profiles are more alike than either is to white skin, and that the expression profile of white skin is more similar to yellow skin than orange skin (fig. S1, Supplementary material online). These results are consistent with a scenario where white and pigment-rich skin differ not only in the reflecting molecules they synthesize but also in the relative abundance of different chromatophore types, while orange and yellow skin differ primarily in the pigments synthesized by erythophores/xanthophores.

### Vertebrate Color and Color-Pattern Genes Are among Differentially Expressed Genes

**Fig. 4.**
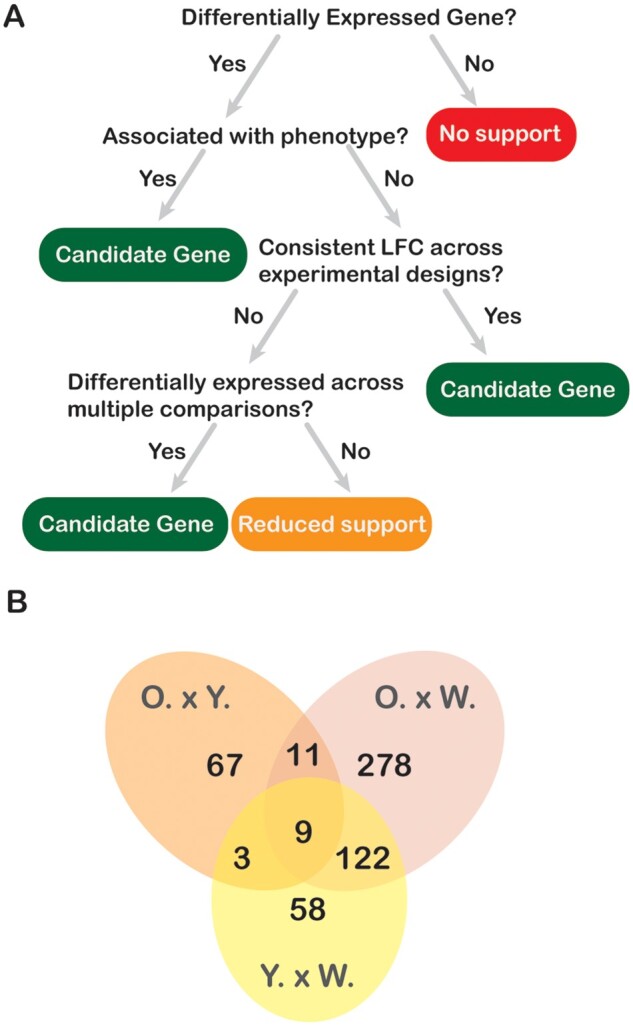
Identifying candidate genes among differentially expressed genes. A. The decision tree we implemented to identify which genes should be considered as candidates for the genetic basis of color and color pattern in the Glowing Ember Bark Anole. The list of all candidate and differentially expressed genes and their log-fold differences in expression across skin comparisons is available at the Supplementary Materials online (supplementary table S5, Supplementary material online; supplementary figs. S8–S11, Supplementary material online). B. Venn Diagram depicting the number of candidate genes shared across pairwise skin comparisons. Notice the large (122) number of candidate genes shared between O. × W. and Y. × W. comparisons, the smaller number of differentially expressed genes exclusive of Y. × W. comparisons relative to O. × W. comparisons, and the overall lower number of differentially expressed genes in O. × Y. comparisons. These results imply a larger similarity in the expression profiles of orange and yellow color, a larger similarity in the profiles of white skin to yellow pigmented skin than orange skin, and a large set of genes that differentiate pigment-rich skin (orange or yellow) from white skin. Key: O. × Y.—orange vs. yellow skin; O. × W.—orange vs. white skin; Y. × W.—yellow vs. white skin.

We reduced our set of 604 differentially expressed genes across all three pairwise comparisons to a pool of candidate genes for color and color pattern using a decision tree based on three criteria (fig. 4*a*; table S3, Supplementary material online). Candidate genes should: i) be functionally linked to vertebrate coloration in prior studies; ii) show consistent log-fold changes in paired and unpaired experimental designs; or iii) be differentially expressed across more than one pairwise skin comparison. Our reasoning for these criteria was as follows: i) given that color and color-pattern genes appear to be conserved across vertebrates (Mills and Patterson 2009), candidate genes should have been linked to color and color pattern in prior studies; ii) if the same molecular mechanisms are responsible for differences in color within and between specimens, then candidate genes should show similar expression patterns across paired and unpaired experimental designs; and iii) if a color or color pattern is determined by the upregulation of a gene relative to its baseline expression level, then candidate genes should be significantly upregulated in a skin color across multiple skin comparisons (e.g., upregulated in orange skin across both orange vs. yellow and orange vs. white comparisons). After applying these three criteria, we reduced our original list of 604 differentially expressed genes to 548 candidate color and color-pattern genes (fig. 4b), of which 93.7% were positively correlated across paired and unpaired experimental designs (fig. S2, Supplementary material online), and 27.0% were differentially expressed across more than one skin comparison.

**Fig. 5.**
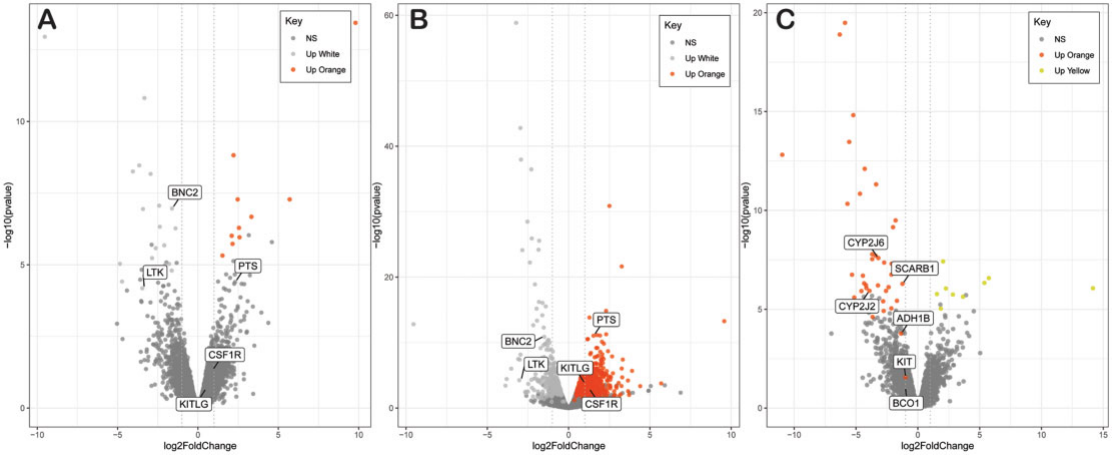
Subset of volcano-plots between log2-fold change and -log10 corrected *P*-values for three comparisons: (A) paired orange vs. white, unpaired orange vs. white, and paired orange vs. yellow. The remaining volcano plot can be found in the Supplementary material online (fig. S12, Supplementary material online). The −log10 corrected *P*-values depicted here were obtained with DESeq2. Results from edgeR and salmon were consistent with those from DESeq2. Each dot represents a gene annotated to the Glowing Ember Bark Anole transcriptome. The vertical dotted light grey lines mar the boundaries of −1 and 1 log2-fold change. Dark grey points represent genes that were not differentially expressed for that skin comparison. Orange, yellow and light gray points represent differentially expressed genes for that skin comparison. Named genes belong to our set of best candidates for color and color pattern. Notice how genes that were not differentially expressed in (A). (paired design) but that were in (B). (unpaired designs) (i.e., *KITLG*, *CSF1R*, *PTS*) show a consistent pattern of upregulation across designs. Notice also that albeit nonsignificant, BCO1 shows an approximate 2-fold upregulation (i.e., log2-fold change ∼ 1) in orange relative to yellow skin.

Because the functional significance of most of these 548 candidate genes for color and color pattern has yet to be tested, we further focused on a subset of these candidate genes that have been functionally verified in studies of model vertebrates. This filtering led to a set of 13 best candidate color and color-pattern genes (table 1; fig 5). These genes include seven genes involved in the maintenance and differentiation of chromatophores (i.e., color-pattern genes: Basonuclin 2 [*BNC2*], Colony Stimulating Factor 1 Receptor [*CSF1R*], Endothelin 3 [*EDN3*], Endothelin Receptor Type B [*ENDRB*], Protoco-Oncogene c-KIT [*KIT*], KIT Ligand [*KITLG*], Leukocyte Receptor Tyrosinase Kinase [*LTK*]), and six genes involved in the synthesis and deposition of pteridines or carotenoids (i.e., color genes: Scavenger Receptor Class B Receptor 1 [*SCARB1*], Beta-Carotene Oxygenase 1 [*BCO1*], Alcohol Dehydrogenase 1B [*ADH1B*], Cytochrome P4502J2 [*CYP2J2*], Cytochrome P450 2J6 [*CYP2J6*], and 6-Pyruvoylterahydropterin Synthase [*PTS*]).

**Table 1:** List of Vertebrate Candidate Color and Color-Pattern Genes

Gene	Protein Name	Color/Pattern	Comparison	Expression
*ADH1B*	Alcohol Dehydrogenase 1B	Color	O. vs Y.	O. > Y.
*BCO1*	Beta-Carotene Oxygenase	Color	O. vs Y.	O. > Y.
*CYP2J2*	Cytochrome P450 Family 2 Subfamily J Member 2	Color	O. vs Y.	O. > Y.
*CYP2J6*	Cytochrome P450 Family 2 Subfamily J Member 6	Color	O. vs Y.	O. > Y.
*SCARB1*	Scavenger Receptor Class B Member 1	Color	O. vs Y.	O. > Y.
*PTS*	6-pyruvoyl Tetrahydrobiopterin Synthase	Color	P. vs W.	P. > W.
*BNC2*	Zinc Finger Protein Basonuclin-2	Pattern	O. vs W. and O. vs Y.	W. > P.
*CSF1R*	Colony Stimulating Factor 1 Receptor	Pattern	O. vs W.	O. > W.
*EDN3*	Endothelin 3	Pattern	O. vs Y.	O. > Y.
*ENDRB*	Endothelin Receptor Type B	Pattern	W. vs Y & O. vs Y.	W. > Y. > O.
*KIT*	KIT Proto-Oncogene, Receptor Tyrosinase Kinase	Pattern	O. vs Y.	O. > W.
*KITLG*	KIT Ligand	Pattern	P. vs W.	P. > W.
*LTK*	Leukocyte Receptor Tyrosine Kinase	Pattern	O. vs W. & O. vs Y.	W. > P

“Gene” and “Protein Name” follow ENSEMBL’s denomination. “Color/Pattern” indicates whether the gene is linked—or inferred to be linked—with either color or color-pattern (see text). “Comparison” indicates in which skin comparison the gene of interest showed a difference in expression. Abbreviations are as follows: O. – orange skin; Y. – yellow skin; W—white skin; P—pigmented skin. We used “P” whenever we found difference between both pigmented skin and white skin, but no difference between orange and yellow skin.

### White and Pigment-Rich Skin Upregulate Different Parts of the Guanine and Pteridine Pathways

We used three types of enrichment analyses to compare expression profiles of color-producing pathways: over-representation tests, competitive enrichment tests, and selfcontained enrichment tests (sensu Goeman and B**ü**hlmann 2007). Over-representation tests tested whether annotated pathways from the KEGG online database (Kanehisa and Goto 2000) were disproportionately represented among differentially expressed genes; competitive enrichment tests tested whether genes from a set defined a priori were differentially expressed as often as genes not in the pathways (i.e., their complement); and selfcontained enrichment tested asked whether at least one gene from an set defined a priori was differentially expressed while accounting for the expected correlated expression patterns for genes from a set. In both competitive and selfcontained enrichment tests, we tested for the enrichment of three pathways that have been previously associated with vertebrate color: the carotenoid pathway (Provitamin A pathway, Waagmeester et al. 2009), the guanine pathway (Higdon et al. 2013), and the pteridine pathway (Ziegler 2003; Braasch et al. 2007).

Due to the presence of guanine platelet-bearing organelles in iridophores and the abundance of iridophores in white squamate skin (Bagnara and Matsumoto 2006), we predicted that white skin would upregulate the guanine-synthesis pathway relative to pigment-rich skin. Both the over-representation and selfcontained enrichment tests supported this prediction, with the over-representation test finding a significant enrichment of genes from the “Nitrogen Metabolism” KEGG pathway (which precedes the synthesis of guanine) in white skin. The selfcontained enrichment test and the barcode plot between log-fold change and local gene enrichment (figs. S3–S5, Supplementary material online) indicated, however, that white skin upregulated part, rather than the entirety, of the guanine-synthesis pathway. This result was further corroborated by the competitive-enrichment test, which did not reject the null hypothesis of enrichment of the guanine-pathway relative to its complement. Pteridines are synthesized from guanosine triphosphate (GTP), which itself is synthesized from guanosine monophosphate (GMP), an important substrate for the synthesis of guanines. Since squamates use pteridines as pigments (Ortiz and Williams-Ashman 1963; Steffen and McGraw 2007; McLean et al. 2017, 2019), we propose that the partial upregulation of the guanine-synthesis pathway in pigment-rich skin could be explained by parts of this pathway associated with the synthesis of GTP being also involved in the synthesis of pteridine in the yellow and orange dewlap skins (Ziegler 2003).

We also predicted that pigment-rich skin would upregulate genes from the pigment synthesizing carotenoid and pteridine pathways relative to white skin. Selfcontained enrichment tests did support our prediction that pigment-rich skin upregulates genes from the pteridine pathway, but the same was not true for the carotenoid pathway (see below). As was the case with the guanine-synthesis pathway, barcode plots and nonsignificant competitive enrichment tests indicated that pigment-rich skin only upregulated part of the pteridine pathway. This could be due to the branched nature of the pteridine pathway, which can be roughly divided into three branches (Ziegler 2003; Braasch et al. 2007; Kim et al. 2019). One branch synthesizes H_4_-biopterin—a molecule that is used, among other processes, in the degradation of phenylalanine and the biosynthesis of neurotransmitters—a second branch synthesizes sepiapterin and its derivatives, and a third branch synthesizes drosopterins (e.g., fig. S3, Supplementary material online). Even though not all enzymes associated with the synthesis of sepiapterins and drosopterins have been described for vertebrates, all three branches overlap at the two-step enzymatic process that synthesizes 6-pyruvoyl-tetrapterin from GTP (Ziegler 2003). Genes from the pteridine pathway upregulated by white skin are either shared across all three branches of the pteridine pathway (e.g., GTP Cyclohydrolase 1 [*GCH1*]), or closely associated with the production of H_4_-biopterin (e.g., Quinoid Dihydropteridine Reductase [QDPR]). Genes upregulated by pigment-rich skin, on the other hand, are either known to play a role in the synthesis of sepiapterins (e.g., *PTS* and Sepiapterin Reductase [*SPR*]) or could play a role in the synthesis of drosopterins—based the function of homologous genes in *Drosophila* (e.g., Glutathione S-transferase omega-1 [*GSTO1*] in Kim et al. 2013; but see below). *PTS*, for example, which is likely a key player in the synthesis of colored pteridines due to its role in converting H_2_-neopterin-TP into 6-pyruvoil-H_4_-pterin, was significantly upregulated in orange skin relative to white skin. Therefore, the parts of the pteridine pathway that were upregulated in pigment-rich skin relative to white skin were consistent with the prediction that orange and yellow skin synthesize more color-reflecting pteridines than white skin.

Contrary to our prediction that the carotenoid pathway would be upregulated in pigment-rich tissues, selfcontained enrichment tests and barcode plots showed that white skin upregulated a large proportion of the carotenoid pathway. White skin significantly upregulated five genes: four genes from the *CYP* family (CYP Subfamily A member 24 [*CYP24A1*], CYP family Subfamily B Member 1 [*CYP1B1*], CYP family Subfamily C Member 1 [*CYP1C1*], and ENSACAG00000009906), and one gene from the aldehyde dehydrogenase family (Aldehyde Dehydrogenase 1 Family Member A2 [*ALDH1A2*]). Pigment-rich skin, on the other hand, upregulated three other genes from the *CYP* family (CYP family 27 Subfamily B Member 1 [*CYP27B1*], CYP family Subfamily 1 Subfamily B Member 8-like [*CYP1B8-like*], CYP family 2 Subfamily W Member 1 [*CYP2W1*]), and one gene from the aldehyde dehydrogenase family (Aldehyde Dehydrogenase 2 Family Member A3 [*ALDH1A3*]). None of the cytochrome P450 genes upregulated in either skin color, however, belongs to the *CYP2J* family that has been linked with the ketolation of yellow xanthophylls (see below). Furthermore, aldehyde dehydrogenases, which were also upregulated in both white and pigment-rich skin colors, are typically associated with xenobiotics breakdown rather than being linked with pigment production in vertebrates (Waagmeester et al. 2009). Since recent findings in integrative studies of reptilian and bird color (Andrade et al. 2019; Gazda et al. 2020) indicate that the deposition and modification of yellow and red carotenoids take place early in the carotenoid pathway, we propose that carotenoid genes differentially expressed between white and pigment-rich skin play roles other than pigment production.

### Orange Skin Upregulates Both Carotenoid and Pteridine Pathway Genes

Because prior chromatographic studies have suggested that anoles use drosopterins to produce red and orange colors and xanthophylls to produce yellow colors (Ortiz and Williams-Ashman 1963; Steffen and McGraw 2007), we predicted that, in comparisons between orange and yellow skin, orange skin would upregulate the pteridine pathway while yellow skin would upregulate the carotenoid pathway. Over-representation and selfcontained enrichment tests showed, however, that orange skin upregulated not only the pteridine pathwa but also the carotenoid pathway. These results were corroborated by barcode and pathway plots (figs. S3 and S6, Supplementary material online), which indicated that orange skin upregulated parts of both pathways relative to yellow skin. The over-representation test found that genes associated with the pteridine and carotenoid pathways were disproportionately represented among differentially expressed genes, including genes annotated to KEGG’s “Retinol Metabolism,” “Folate Biosynthesis,” and “Metabolism of Xenobiotics by Cytochrome P450” pathways. Accordingly, differential expression tests found that orange skin upregulated four genes from the carotenoid pathway (see below).

Although a higher expression of carotenoid pathway genes in orange skin was unexpected based on prior histological and chromatographic studies in anoles (Ortiz and Williams-Ashman 1963; Steffen and McGraw 2007), higher concentrations of both carotenoids and pteridines in orange skin relative to yellow skin have been reported in the distantly related Australian frilled-neck lizard (*Chlamydosaurus kingii*, McLean et al. 2019). In our comparisons, orange skin upregulated almost all of the pteridine pathway, with the exceptions of *PTS* and genes we and other authors tentatively assigned to the synthesis of drosopterins based on homology to *Drosophila* sequences (Braasch et al. 2007; McLean et al. 2017). Similar to our results, for example, McLean et al. (2017) also found that one of these candidate drosopterin synthesis genes (*TXNDC15*) showed lower expression in orange skin relative to yellow skin, despite chromatographic data identifying drosopterins deposited in the skin of their study species. Therefore, is not clear based on transcriptomic data alone whether drosopterins are playing a role in orange or red coloration in the Glowing Ember Trunk Anole.

Evidence is accumulating that pigments synthesis and deposition can vary widely even among closely related vertebrate species (e.g., Twomey et al. 2020). Recent chromatographic studies in squamates (Stuart-Fox et al. in press) indicate that squamates can compensate for a lower availability of dietary carotenoids and maintain a similar spectrophotometric profile by upregulating the synthesis of pteridines. We, therefore, suggest three explanations for our observations: i) a set of genes different from the one we and others hypothesized, based on homology to sequences in *Drosophila*, to be responsible for the synthesis of drosopterins could be responsible for this process in squamates; ii) the synthesis of drosopterins takes place elsewhere in the body, with drosopterins being transported to and deposited in the skin postsynthesis; and iii) long-wavelength reflecting ketocarotenoids, either with or independently from drosopterins, are responsible for differences between orange and yellow skin colors in the Hispaniolan Bark Anole.

### A Model for the Regulation of Carotenoid-Based Color Production in Anoles

Like other vertebrates, anoles are unable to synthesize carotenoids de novo and must obtain them from their diets (Widjaja-Adhi and Golczak 2020). To use carotenoids as pigments, anoles must first absorb these molecules through their digestive tract, transport them to the dermis through the bloodstream (likely with the aid of lipoproteins; Widjaja-Adhi and Golczak 2020), move them into chromatophores, and potentially modify these dietary carotenoids within chromatophores to reflect the observed wavelength. In mice, carotenoid uptake from the bloodstream is regulated by a negative feedback loop that involves the production of retinoic acid via cleavage of carotenoids by an oxygenase, typically *BCO1* (Widjaja-Adhi et al. 2015). In birds, hair follicles take in xanthophylls from the blood and either directly deposit them in yellow feathers, or ketolate them via a cytochrome P450 monooxygenase (*CYP2J19*) prior to depositing them in red feathers (Lopes et al. 2016; Mundy et al. 2016). Below, we outline, given our results, how a similar feedback mechanism to that found in mice might be regulating the synthesis of orange and yellow carotenoid-based pigments in anoles.

Anole xanthophores/erythrophores likely use a protein from the scavenger receptor class B (*SCARB*) family to import carotenoids from the bloodstream (Widjaja-Adhi et al. 2015). Thus far, three *SCARB* proteins (*SCARB1*, *SCARB2*, and *SCARB3*) are known in vertebrates, two of which have been identified as candidate genes for color differences: *SCARB1* and *SCARB3* (Connelly and Williams 2004; Shen et al. 2018). *SCARB1* has been linked to yellow coloration in canaries, where individuals homozygous for an abnormally spliced allele have white rather than yellow feathers (Toomey et al. 2017); *SCARB3* was identified as a candidate color gene on anoles in a thesis work that used transcriptomic comparisons between pink dewlap, white belly, and green back skin in *Anolis carolinensis*, as well as in scans for genomic differentiation between populations of *Anolis marmoratus* with orange and blue back skin (Crawford 2013). Our transcriptomic comparisons identified *SCARB1*, but not *SCARB3*, as a candidate gene for orange coloration in anoles by finding that orange skin significantly upregulated this gene relative to yellow skin.

Once carotenoids are absorbed by a xanthophore/erythrophore, they can be modified and used in multiple processes, including the synthesis of retinol (Waagmeester et al. 2009). *BCO1* has long been known to play a role in the synthesis of retinol by catalyzing the oxidative cleavage of beta-carotene into two retinal molecules (Widjaja-Adhi et al. 2015; Harrison and Kopec 2020). Recently in mice, *BCO1* was also shown to be part of a negative feedback look that regulates the production of vitamin A (Widjaja-Adhi et al. 2015). Specifically, *BCO1* converts beta carotene into retinal, which is then converted into retinoic acid. This retinoic acid induces the expression of a homeobox transcription factor that regulates the synthesis of *SCARB1*. We found that orange skin strongly (log fold change _**∼**_1), albeit not significantly, upregulated the synthesis of *BCO1* relative to yellow skin. In addition, orange skin significantly upregulated a second gene associated with the production of retinoic acid, *ADH1B* (Waagmeester et al. 2009), which could also take part in this negative feedback loop controlling intake of carotenoids from the bloodstream.

If anoles use ketocarotenoids to produce red or orange colors, and if ketocarotenoids are not available through dietary intake, the next step would be for anoles to ketolate the yellow xanthophylls they obtain from their diet into orange or red colored ketocarotenoids. Ketolation is linked to the cytochrome P450 family (*CYP*) of monooxygenases across eukaryotes (Mundy et al. 2016; Twomey et al. 2020). The enzyme responsible for ketolation in birds and turtles (which encompass archosaurs, the sister clade of lepidosaurs [tuataras + squamates]), was recently identified as *CYP2J19*, a gene that is not present in the anole genome (Lopes et al. 2016; Mundy et al. 2016; Twyman et al. 2016). Given that *CYP* is used to ketolate carotenoids in taxa as divergent as anurans and birds, and that archosaurs use *CYP2J19* to perform this task, the process of ketolation implied by transcriptomic and chromatographic studies in Australian Lizards (McLean et al. 2017; McLean et al. 2019; Stuart-Fox et al. 2021) as well as our own is likely performed by another enzyme in the *CYP* family.

Given the negative feedback loop controlling the synthesis of *SCARB1* in mice, and the concomitant higher expression of *ADH1B*, *SCARB1*, and *BCO1* in orange skin relative to yellow skin, we hypothesize that a negative-feedback system such as the one described in mice also occurs anoles. If this is true, the synthesis of *SCARB1* is regulated by the breakdown of carotenoids into retinoic acid by an oxygenase like *BCO1* or *ADH1B*. This negative-feedback system, along with the differential upregulation of two *CYP2J* genes in orange skin relative to yellow skin in the Glowing Ember Trunk Anole, and previous chromatographic studies in other anoles, led us to propose two nonexclusive mechanisms which could explain the color differences between orange and yellow dewlaps in this taxon. First, in a ketocarotenoid-free scenario, we propose that orange colors could be produced by a combination of orange or red drosopterins along with a yellow xanthophylls and sepiapterins—which when in higher concentration shift the chromatophore’s peak wavelength reflection from yellow toward longer orange or red wavelengths. Alternatively, we propose that ketocarotenoids could play a role in producing orange colors along with sepiapterins and xanthophylls. In this second mechanism, xanthophylls scavenged by chromatophores from the bloodstream would undergo ketolation within the chromatophore. This second mechanism also allows for the combined action of drosopterins and ketocarotenoids in producing orange or red colors. If this second mechanism holds true, then it is likely that one of the two *CYP2J* genes we found to be significantly upregulated in orange skin, *CYP2J2* and *CYP2J6* (fig. S7, Supplementary material online), could be responsible for ketolase activity in anoles.

### Zebrafish Color-Pattern Genes are Differentially Expressed between Iridophore- and Xanthophore/Erythrophore-Rich Ski*n*

Our results supported our prediction that different skin colors would upregulate color-pattern genes associated with the development and maintenance of different chromatophores: iridophores in white skin and xanthophores/erythrophores in pigment-rich skin. Seven of our 13 best candidate genes for anole color-pattern have been functionally linked to color pattern in zebrafish (table 1) (reviewed in Singh and N**ü**sslein-Volhard 2015; Irion et al. 2016; Patterson and Parichy 2019). These genes are responsible for the migration and differentiation of neural crest cells, as well as the maintenance of specific types of chromatophores postdifferentiation in zebrafish.

Four of the seven zebrafish color-pattern genes that exhibit significant expression differences in our transcriptomic comparisons are functionally linked to iridophore differentiation and maintenance in zebrafish (*BNC2*, *LTK*, *EDNRB*, and *END3*). *BNC2* mutants show lower differentiation and higher mortality rates of iridophores (Lang et al. 2009), *LTK* mutants exhibit lower differentiation, proliferation, and survival of iridophores (Fadeev et al. 2016), *EDNRB* mutants have their iridophore differentiation disrupted during metamorphosis (Parichy et al. 2000), and *EDN3* mutants have their iridophore proliferation disrupted postdifferentiation (Spiewak et al. 2018). All four of these genes are significantly upregulated in iridophore-rich white anole skin.

The other three zebrafish color-pattern genes identified in our study are linked to xanthophore/erythrophore or melanophore maintenance and differentiation in zebrafish. Both orange and yellow dewlap skin upregulated *CSF1R* relative to white skin, while orange skin upregulated *KIT* and *KITLG* relative to both yellow and white skin. In zebrafish, the migration of xanthophore/erythrophore precursors, as well as their maintenance postdifferentiation are disrupted in *CSF1R* mutants (Parichy et al. 2000), while the migration of melanophore precursors, as well as the maintenance of melanophores postdifferentiation are disrupted in *KIT* and *KITLG* mutants (Parichy et al. 1999; Dooley et al. 2013). *CSF1R* is upregulated in pigment-rich orange or yellow dewlap skin, which is expected to contain more xanthophores/erythrophores than white belly skin, while *KIT* and *KITLG* are upregulated in orange skin, which shows lower brightness and contains more melanophores than either yellow or white skin (Ng et al. 2013, PdM pers. obs.).

This set of seven best candidate genes for color pattern represent more than one quarter of the 26 genes linked in a recent review to zebrafish color pattern (Patterson and Parichy 2019). If we assume the expression of each color-pattern gene to be independent, we should expect to find at most one (_**∼**_0.8396) of these genes among our set of 604 differentially genes. In addition, the expression patterns we observed across these seven color-pattern genes are consistent with expression patterns predicted by previous functional research in zebrafish, where, for example, iridophore development and maintenance genes being more expressed in white skin, and xantophore/erythrophore development and maintenance gens being more expressed in orange or white pigment-rich skin (Patterson and Parichy 2019). These results are in agreement previous research on model organisms (Mills and Patterson 2009) that suggested that genes responsible for chromatophore development, differentiation and maintenance are conserved across vertebrates, making the color-pattern genes we identify herein excellent candidates for future functional studies on the genetic basis of color-pattern in anoles.

## Conclusions

Through transcriptomic comparisons, we found significant differences in the expression profiles of white, orange, and yellow skin sampled from the Glowing Ember Trunk Anole. White skin upregulated genes from the guanine pathway as well as genes functionally associated in zebrafish with the development and maintenance of iridophores. Meanwhile, pigment-rich dewlap skin upregulated and differentially expressed genes from both the pteridine carotenoid pathways. Although chromatographic studies of anole skin previously suggested that orange and yellow coloration result primarily from pteridines (Ortiz and Williams-Ashman 1963; Steffen and McGraw 2007), the upregulation of both the pteridine and carotenoid pathways in orange tissue relative to yellow and white tissues supports recent transcriptomic and chromatographic that indicated that both carotenoids and pteridines can be involved in the production of orange and red colors in squamates (McLean et al. 2017, 2019; Stuart-Fox et al. 2021). Accordingly, orange skin upregulated genes previously linked to the regulation of carotenoid intake from the bloodstream in mice (*BCO1, SCARB1*, *ADH1B*; Widjaja-Adhi et al. 2015), genes from the *CYP* family of monooxygenases that ketolate xanthophylls in birds (Toews et al. 2017), and genes responsible for the development and maintenance of melanophores and erytrhophores/xanthohores in zebrafish (*CSF1R*, *KIT*, *KITLG*; Patterson and Parichy 2019). These results led us to propose two nonexclusive mechanisms for regulating the intake of carotenoid-based color in the Glowing Ember Trunk Anole.

Our results also corroborate the hypothesis that not only genetic pathways responsible for producing color-reflecting molecules, such as carotenoids and pteridines, but also those pathways responsible for the development, differentiation, and maintenance of chromatophores are conserved across vertebrates. Furthermore, the expression of color genes between differently colored patches of skin adds to a growing literature that indicates that squamates can use different combinations of structural and pigmentary mechanisms to reflect similar colors. The same, however, appears to not be necessarily true for color-pattern genes, which showed the same expression patterns in the Glowing Ember Trunk Anole as would be expected based on our current knowledge from zebrafish.

Even though RNA sequencing is not without its shortcomings, it has become a very powerful tool to characterize and quantify expression patterns throughout the transcriptome (Ozsolak and Milos 2011; Hrdlickova et al. 2017). RNA-seq has become a common intermediate discovery step of causal genes in evolutionary biology, given differentially expressed genes provide lists of candidates for functional studies, which are key to linking genotype with the proposed phenotype (Van den Berge et al. 2019). This is particularly true in emerging model systems like anole lizards, for which CRISPR-Cas9 has recently been established (Rasys et al. 2019). Our study, therefore, not only characterizes the expression profiles of orange, white, and yellow skin in the Glowing Ember Trunk Anole, but also provides a list of candidate color and color-pattern genes to be functionally verified in anoles, a key first step in unveiling the genetic basis of squamate color and color-pattern.

## Materials and Methods

### Sample Collection, RNA Extraction, and Sequencing

We collected seven males from a single locality in southern Dominican Republic, Barahona Peninsula, in January 2019: two individuals with fully orange dewlaps, two individuals with fully yellow dewlaps, and three individuals with bicolored dewlaps (fig. 3*b*). We excised 14 skin samples from across three regions of the body, with at most one sample per region per animal. In total, we obtained four samples from white belly, five from orange dewlaps, and five from yellow dewlaps (table S2, Supplementary material online).

We humanely killed specimens following applicable institutional guidelines for animal care and welfare under the University of Kansas IACUC protocol Animal Use Statement (AUS) 208-03, and sampled tissues immediately postmortem. We followed Macedonia et al.’s (2000) approach for excising dewlap skin (Supplementary material online). We homogenized samples using a Mini-Beadbeater 96 (Biospec Products) with a 3 mm Tungsten Carbide bead for 30 s at 2400 rpm and extracted total RNA using the Quick RNA Miniprep kit (Zymo Research) following the manufacturer’s protocol.

We sent total RNA extractions to the University of Kansas’ Genome Sequencing Core (KU-GSC) for library preparation with the New England BioLabs Next Ultra II Direction mRNA kit. Prior to pooling, the KU-GSC verified each sample’s quality by quantifying its concentration with a Qubit 2.0 Fluorometer (Invitrogen) RNA HS Assay Kit and assessing its integrity by running it on an Agilent Tapestation 2200 with a High Sensitivity RNA chip (table S2, Supplementary material online). After all samples passed the quality control steps, the KU-GSC pooled each individually barcoded sample into a single pool that they sequenced twice to obtain an average of _**∼**_29 million reads per sample. This pool was sequenced once through a High Output and once through a Mid-Output lane on the Illumina Nextseq platform with 75 bp paired end reads.

### Reference Transcriptome Assembly

To assemble the Glowing Ember Trunk anole transcriptome, we selected a single specimen with RIN > 9 scores across all three skin colors (table S2, Supplementary material online). We implemented the Oyster River Protocol (ORP) (MacManes 2018), which uses orthology of transcripts between Trinity and Oases (Grabherr et al. 2011; Schulz et al. 2012), to generate a consensus *de novo* assembly for the Glowing Ember Trunk anole skin.

Prior to running the ORP, we removed potential contaminants by querying each sample against a custom contaminant database using bbduk v.38.73 (Bushnell 2020). We downloaded contaminant data from two sources: i**)** the Silva rRNA database (Quast et al. 2013); and ii**)** the NCBI genome database (Pruitt et al. 2005). We list all contaminant genomes we used when running bbduk in the Supplementary material online (supplementary table S4, Supplementary material online). We assured that no contaminant persisted after cleaning with bbduk by running FastQ Screen (Wingett and Andrews 2018).

To annotate the de novo transcriptome, we used a multistep process based on de novo and reference-based annotations. We started the process by running the de novo and reference-based steps in parallel. We de novo annotated using the Sequence Massive Annotation by Modules v2 (sma3s) (Casimiro**‐**Soriguer et al. 2017), and we reference-based annotated by aligning *A. distichus* transcripts to the well-annotated *Anolis carolinensis* transcriptome (from ENSEMBL, Yates et al. 2020) using blat (Kent 2002). Next, we compared both *de novo* and reference-based annotations and identified transcripts exclusively annotated by sma3s, adding them to the reference-based annotation. To reduce the redundancy of transcripts in this hybrid annotation, we then clustered transcripts with a similarity score _**≥**_ 80% using CD-HIT-EST (Huang et al. 2010). Finally, we identified which annotated transcripts had open reading frames (ORF) using GeneMarkS-T (Tang et al. 2015). The final annotated Glowing Ember Trunk Anole transcriptome assembly consisted of 51,259 transcripts assigned to 18,734 putative genes, 13,522 of which contained ORFs. We assessed the quality of our skin transcriptome by aligning it to the Benchmarking Universal Single-Copy Orthologs (BUSCO) vertdb10 database (Seppey et al. 2019). The final assembly encompassed 82.5% of the BUSCO vertdb10 genes, 71.3% of which were complete. To further assess the quality of our assembly, we estimated the percent identify of our candidate genes (see below) against the *A. carolinensis* transcriptome. On average, we found a percent identify of 90.47% between our assembled transcripts and the transcripts annotated in the *A. carolinensis* transcriptome.

### Bioinformatics

We assessed each sample’s raw read quality with FastQC v.0.11.9 (Wingett and Andrews 2018) and visualized the results across samples with multiqc v.1.8 (Ewels et al. 2016). We removed adapter contamination and low quality sequences (phred < 20 across 4 bp windows, total sequence length < 40 bp) using Trimmomatic v.0.39 (Bolger et al. 2014). Like we did for reads used in the transcriptome assembly, we removed potential contaminants by querying sample reads against a custom set of contaminants using bbduk v.38.73 (Bushnell 2020). Lastly, we corrected for random sequencing error in raw reads using the k-mer based method Rcorrector (Song and Florea 2015), and checked the quality of the filtered data once more using FastQC, FastQ Screen, and multiqc.

### Data Visualization

We visualized differences in expression patterns between skin colors by performing a principal component analysis (PCA) on rlog normalized expected read counts with the “prcomp” function from R (R Core Team 2013). We estimated read counts across all pairwise tissue comparisons and experimental designs with salmon (Patro et al. 2017), and rlog transformed these counts with “DESeq2” (Love et al. 2014).

### Identifying Candidate Genes for Color and Color Pattern

We identified genes and gene sets responsible for phenotypic differences across squamate skin with two complementary methods: differential expression and gene set enrichment analyses.

#### Differential Expression Analyses

To identify differentially expressed genes, we implemented three differential expression pipelines across an unpaired and a paired experimental design (see Supplementary material online for details). In the unpaired design, we used a single sample from each specimen; in the paired design, we used pairs of samples from each specimen (i.e., each specimen contributed with one sample from each skin color). To statistically control for idiosyncratic expression patterns shared by paired samples, we used specimen identification as a fixed factor when fitting the generalized linear models for differential expression in paired designs.

Given that the list of differentially expressed genes from distinct pipelines commonly shows only partial overlap, we chose to combine information from the three pipelines to identify a gene as differentially expressed. This partial overlap is due to peculiarities of each pipeline such as the read alignment software, the read count normalization, and the variance shrinkage approach implemented in the differential expression pipeline (Zhang et al. 2014; Costa-Silva et al. 2017). After performing preliminary comparisons across multiple software combinations, we restricted our analyses to three differential expression pipelines consisting of two read count and three differential expression software: salmon + DESeq2, salmon + edgeR and kallisto + sleuth (Robinson and Oshlack 2010; Love et al. 2014; Bray et al. 2016; Patro et al. 2017; Pimentel et al. 2017). Prior to running differential expression analyses, we converted expected transcript-wise read counts into expected gene-wise read counts using the R package tximport (Soneson et al. 2016). We ran kallisto, sleuth, edgeR, DESeq2, and tximport in R v.3.6.3 (R Core Team 2013).

Once we obtained gene-wise *P*-values for each pipeline within each experimental design, we used Fisher’s Combined Test (Fisher 1934) to identify which genes showed a consistent pattern of differential expression across all three pipelines. We considered a gene as candidate if it had a log_2_ fold change _**≥**_ 1 (i.e., at least a 2-fold difference in expression) and a Fisher’s combined test false discovery rate _**≤**_ 0.05. We implemented Fisher’s Combined Test with the “fisher, method” function from the R package “metaseqR” (Moulos 2020).

Lastly, we identified a differentially expressed gene as a candidate gene if it (fig. 4*a*): i) has been functionally linked to color or color pattern in other vertebrate taxa; ii) was consistently differentially expressed across more than one skin comparison (e.g., upregulated in orange skin relative to both yellow and white skin); or iii) showed consistent log-fold changes in expression across experimental designs for a given skin comparison. We tested for (iii) by estimating the correlation between log-fold changes across paired and unpaired experimental designs using R package “Rmisc” (Harrell 2020).

#### Gene Set Enrichment Analyses

We focused our gene set enrichment analyses on gene sets hypothesized to be responsible for color and color pattern differences across yellow, orange, and white skin. White colors are hypothesized to be the product of a coherent scattering of light by guanine-platelets deposited in iridophores, while yellow and orange colors are hypothesized to be reflected by pteridines and/or carotenoids deposited in xanthophores/erythrophore (Bagnara and Hadley 1973). Therefore, following McLean et al. (2017), we tested for the enrichment of pathways associated with the synthesis of guanines, pteridines, and carotenoids (table S5, Supplementary material online). The “guanine synthesis” pathway included the enzymatic precursors for the production of guanine from phosphoribosyl pyrophosphate (Higdon et al. 2013); the “pteridine synthesis” pathway included genes from the tetrahydro-biopterin biosynthesis module, as well as genes responsible for the synthesis of drosopterins and sepiapterins (Ziegler 2003; Braasch et al. 2007); and the “carotenoid synthesis” pathway included genes from the retinol (vitamin A) metabolism (Waagmeester et al. 2009).

We used gene set enrichment analyses to test if genes belonging to each of these three gene sets (Goeman and B**ü**hlmann 2007): i**)** were disproportionately represented among differentially expressed genes (i.e., over-representation test); ii**)** were differentially expressed as frequently as genes not in the gene set (i.e., competitive enrichment test); or iii**)** contained at least one differentially expressed gene (i.e., selfcontained enrichment test). We performed gene set enrichment analyses only for the unpaired experimental design. We implemented gene set enrichment analyses with three software: “enrichKEGG,” from the R package “clusterProfiler” v3.0.4 (over-representation test; Yu et al. 2020), and “fry” (selfcontained enrichment test; Wu et al. 2010), as well as “camera” (competitive enrichment test, Wu and Smyth 2012) from the R package edgeR. Prior to performing the over-representation test, we used KEGG’s Online Blast KEGG Orthology and Links Annotation (blastKOALA; Kanehisa et al. 2016) to align the differentially expressed transcripts with ORFs against the KEGG GENES database.

## Supplementary Material

evab110_Supplementary_Data
