# Supplementary material for: Transcriptomic Analysis of Skin Color in Anole Lizards
Source: Genome Biol Evol. 2021 May 14;13(7):evab110. doi: 10.1093/gbe/evab110 (PMC8290120; doi:10.1093/gbe/evab110)
Supplement: evab110_Supplementary_Data [file evab110_supplementary_data.zip › de_Mello_etal_2020_Dewlap_Color_Comparative_Transcriptomics_Supplementary_Materials_OUP.pdf]

Supplementary Materials and Methods

Tissue Sampling and RNAseq Extraction Protocol

We sampled white skin by excising a 1x1 mm patch of belly skin midway through the specimen's snout-vent-length. We sampled orange and yellow skin by excising the entire dewlap with scissors and removing the hyoid cartilage with a forceps. If the specimen had a bicolored dewlap, we cut the yellow margin of the dewlap from the orange gular region. After excision we rinsed all tissues with molecular grade water (Fisher), and placed each skin patch in separate tubes with RNAlater (Invitrogen). We sterilized dissection tools between excisions to avoid cross-contamination. Throughout the process, we kept the RNAlater-filled tubes in an ice bucket. After we excised all tissues we flash froze the RNAlater filled sample tubes in liquid Nitrogen and kept samples in -80 °C freezer until homogenization.

We homogenized each skin sample in RNA Shield Lysis Tubes (Zymo Research) using a Mini-Beadbeater 96 (Biospec Products) for 30 s at 2400 rpm. After homogenization, we quickly spun down samples in a cold centrifuge at 4 °C and flash froze the homogenized tissues in liquid nitrogen. We kept samples frozen at -80 °C until extraction.

The extraction protocol we implemented performs two steps to remove gDNA contaminants, in addition to the column-based RNA extraction. The protocol is as follows. Using a laminar flow cabinet to prevent contamination, we allowed samples to thaw, and added and mixed 1 volume of RNA Lysis Buffer (1:1) to each sample. We then centrifuged the samples for 1 minute at room temperature, and collected and transferred the supernatant to a Spin-Away Filter placed on top of a collection tube. Next, to remove gDNA contamination, we centrifuged this supernatant through a Spin-Away filter for 1 minute. We then added 1 volume of 95% ethanol to the collected flow-through, flicking the tube to ensure thorough mixing. We then transferred this solution to a Zymo-Spin IIICG column placed on top of a new collection tube, centrifuging it for 30 s, but now discarding the flow-through. Next, to further remove gDNA, we treated the sample with DNase I by washing the column with 400 µl RNA Wash buffer, centrifuging for 30 s, and discarding the flow-through. We then added 80 µl of DNase I + DNA digestion buffer (1:15) to the column, incubated at room temperature for 15 min and centrifuged for 30 s, discarding the flow-through. Then we performed two sequential washes, centrifuging 400 µl of RNA Prep Buffer and 700 µl of RNA Wash Buffer through the column for 30 s each, which we we followed with a final wash, centrifuging 400 µl of RNA Wash Buffer for 2 min to ensure complete removal of buffer, discarding the flow-through after each wash. Finally, we added 100 µl of DNase/RNase-Free water to the column matrix, centrifuged for 30 seconds, collected flow-through and stored extraction at -80 °C until library preparation. We performed all centrifugation steps at 10,000 x g.

Aligning Reads and Estimating Expected Read Counts

Read alignment usually consists of two mains steps: building an index and aligning reads or k-mers to this index. Aligning and quantifying RNAseq data is a complex process due to, among other things, the high variation in the number of aligned reads per gene, the small sample sizes, and the existence of introns, pseudogenes and multimapping reads (Deschamps-Francoeur *et al.*, 2020). As a consequence, RNAseq pipelines attempt to correct for these and other sources of noise mathematically. Typically, RNAseq quantification software incorporate this inherent uncertainty in their estimates by using a likelihood and/or Bayesian approach to produce estimated counts for each transcript from the aligned reads (Patro *et al.*, 2017; Bray *et al.*, 2016; Li and Dewey, 2011). In addition, by implementing likelihood and/or Bayesian methods one can incorporate factors that could otherwise bias the estimation process, such as differences in the effective length of sequenced transcripts, sequencing biases in GC-content, and positional biases.

Since quantification software diverge on how they align and quantify read counts, we performed a preliminary comparison of three alignment and quantification pipelines to assess whether the estimated read counts per sampled varied across software. We focused on two approaches that estimate transcript read counts after mapping reads to a transcriptome: The first approach uses bowtie2 (Langmead and Salzberg, 2012) to align reads to a reference, and RSEM (Li and Dewey, 2011) to estimate read counts through a maximum-likelihood count estimator; the second approach relies on single software to perform both the 'pseudo-alignment' and the estimation of read counts from aligned reads sequentially.

In our first approach we used bowtie2 to align reads, and RNA-seq by Expectation Maximization (RSEM) to estimate read counts. bowtie2 combines a full-text minute transcriptome indexing and dynamic programming to align reads to a reference. It starts by using a Burrows-Wheeler transform based on full-text minute space indexing to index a reference. Next, bowtie2 extracts 'seeds' (16base pair [bp] long each, in 10bp windows) from sequenced reads and their reverse complement and aligns them to the reference index in an ungapped fashion. bowtie2 keeps track of quality scores when performing the alignments, allowing for mismatches between read and reference. Once a set of priority alignments are identified, bowtie2 uses dynamic programming to extend alignments while accounting for indels between reads and references. RSEM then uses the mapping number and position information from bowtie2's SAM/BAM output to estimate the expression levels for each transcript. Given the read length, quality score, and sequence, RSEM estimates the parent transcript, length, start position and orientation for each read. RSEM parses out multi-mapped reads by using alignment information from all valid reads in order to restrict the possible positions from where each read could be derived. Then, RSEM uses a maximum-likelihood (MK) approach through an expectation-maximization (EM) algorithm to calculate expression levels for each transcript. Lastly, RSEM uses a Bayesian version of its likelihood with Gibbs sampling to obtain 95% confidence intervals and posterior mean estimates of counts in addition to its maximum likelihood estimate.

The second approach, which we implemented using software from two research groups - kallisto (Bray *et al.*, 2016) and salmon (Patro *et al.*, 2017) - has a single software perform both the pseudo-alignment and the transcript quantification steps. kallisto and salmon align reads to transcripts by aligning read k-mers, instead of full reads, to a transcriptome built index. kallisto uses an approach called 'pseudo-alignment' that identifies which transcript a read is likely to have have originated from, without pinpointing exactly to which position in the transcript the read aligns. Pseudo-alignment works in two main steps. First it uses transcriptome k-mers to build a search index based on a de Brujin graph structure; then, it intersects read k-mers with the search index to identify from which transcript each read originated from. Due to its de Brujin based transcriptome index, kallisto is able to align most reads with

just a fraction of its constituent k-mers, massively increasing processing speed. Consequently, kallisto is able to perform bootstrap replicates of transcript count estimates, which are used downstream by sleuth (the differential expression software developed by the same research group) when parsing biological and inferential variance in the results.

Like kallisto, salmon maps read k-mers to a search index while dropping redundant k-mers to speed up computational time. However, instead of pseudo-mapping, salmon uses RapMap's (Srivastava *et al.*, 2016) 'quasi-mapping' approach for read alignment. A quasi-mapping search index is the combination of a suffix array built from the transcriptome, and a hash table that maps each k-mer from the transcriptome to the suffix array. Thus, by building a suffix array and a hash table, salmon is able to keep track of both positional information and orientation for the aligned reads, which are used downstream in its likelihood calculations. Positional information can also be obtained from kallisto's pseudo-aligned reads *post hoc*. In kallisto, pseudo-alignments are performed *a priori*, and positional information is not used in kallisto's likelihood calculations to estimate read counts. salmon's likelihood read count estimates, on the other hand, explicitly account for parameters not considered by kallisto, such as read orientation and position as well as transcript effective length. Furthermore, salmon incorporates bias models for GC content and sequence-specific bias in its effective length estimations using a combination of Bayesian and maximum likelihood approaches to quantify effective read counts and transcript per million values for each transcript.

By performing preliminary runs with different alignment and quantification pipelines we found that the number of estimated read counts identified by the three alignment + read estimation pipelines (kallisto (Bray *et al.*, 2016) and salmon (Patro *et al.*, 2017), and bowtie2 + RSEM (Langmead and Salzberg, 2012; Li and Dewey, 2011)) were significant and highly correlated ($r \geq 0.95$, $p \leq 0.05$; Fig. S2). These results suggested that most differences between differential expression pipelines were likely to occur during the differential expression estimation step (see Supplementary Material online). Consequently, we restricted our analyses to the kallisto + sleuth and salmon + DESeq2 or EdgeR pipelines.

Differential Gene Expression Software

Differential expression analysis software are developed to deal with three main issues: small sample sizes, read count heteroscedasticity, and multiple testing. All three software applications we used model normalized reads counts with a generalized linear model. The models used by these three applications differ, however, in how read count dispersion is partitioned, and whether they test for differential transcript or gene expression. We detail the implementation of each software application below.

edgeR

The empirical analysis of differential gene expression in R (edgeR) (Robinson *et al.*, 2010) can be summarized in three steps: (1) normalization of read counts; (2) usage of shared information across genes to estimate dispersion and log fold changes of expression coefficients for each gene; and (3) testing for differential gene expression. edgeR normalizes read counts using the trimmed mean of M-values (TMM) method (Robinson and Oshlack, 2010). TMM normalization is implemented to adjust for both sequencing depth, and library composition differences between samples. TMMs steps are as follows: (i) removal of untranscribed genes in both samples; (ii) selection of a reference sample for normalization ; (iii) filtration of genes that have zero reads in either the reference or the scaled samples, or that are outliers for either log2 ratios between reference and scaled sample or log2 geometric means of scaled read counts ; (iv) calculation of the weighted trimmed mean of the log2 ratios between pairs of samples ; and (v) center scaling of factors around 1. Following edgeR's recommendations, we removed all genes with less than 1 count per million (TMM/$1\hat{6}$) in 2 or more samples.

edgeR assumes that the variation between gene read counts across samples can be partitioned into a biological and a technical components, and that a gene-wise biological coefficient of variation can be estimated using information from across sequenced genes (Robinson *et al.*, 2010). To account for small samples sizes and heterogeneous library composition across samples, edgeR fits a negative binomial generalized linear model to the TMMs using an Empirical Bayes approach. The negative binomial generalized linear model uses as input a design matrix and three parameters: normalized read counts, dispersion estimates, and expression coefficients. The empirical Bayes method uses a weighted prior to estimate the posterior likelihood of the dispersion estimate and to shrink (i.e. statistically approximate gene-wise dispersion towards a hypothesized global dispersal) gene-wise expression values. The weighted prior is estimated as the maximum likelihood estimate for the common dispersion across all genes in a sample multiplied by a coefficient which can be set up by the user or, as we have done, be left at default. Lastly, posterior likelihoods of dispersion estimates are calculated using an adjusted profile likelihood - which is a penalized log-likelihood. Once dispersion values have been estimated, edgeR uses a modified Fisher's exact test to test the null hypothesis of no-difference (i.e. equal expression) in dispersion between groups.

DESeq2

DESeq2 also uses a negative binomial generalized linear model to fit expression data. The DESeq2 approach can be broadly divided in three steps: (1) normalization of reads, (2) shrinkage of dispersion estimation, and (3) hypothesis testing. DESeq2 differs from edgeR in how reads are normalized, the calculation of the prior in the empirical Bayes approach, and in the shrinkage of log fold estimates and dispersion coefficients prior to hypothesis testing. As a first step, DESeq2 normalizes reads using the Median-of-Ratios approach (Anders and Huber, 2010). This approach accounts for both differences in library size and composition. The scaling factors for each sample used in the Median-of-Ratios approach are obtained in 3 steps: (i) calculation of the average of the ln (natural log) read counts across all samples, discarding those with zero counts in at least one sample; (ii) for each gene, calculation of the ratio between each sample's read count and the average ln read count; (iii) for each sample, calculation of the scaling factor as the antiln (anti natural log) of the median of the read count ratios for each sample; (iv) normalization by dividing each gene's summarized read count by its sample scaling factor.

DESeq2 then uses a slightly distinct empirical Bayes approach from edgeR to shrink the dispersion estimates. DESeq2 starts by fitting a negative binomial generalized linear model without a log fold change prior to the normalized reads to obtain a gene-wise maximum likelihood dispersal estimate. Then, DESeq2 regresses gene-wise dispersion estimates on

the means of the normalized counts using a gamma-family generalized linear model to obtain the regression's residuals. DESeq2 obtains the empirical prior estimate of the dispersion for the empirical Bayes treatment by matching the distribution of log residuals to a density of simulated log residuals that follow a chi-squared distribution. Next, DESeq2 obtains the final, shrunken, maximum *a posteriori* dispersal parameter estimates by fitting the negative binomial distribution to the summarized read counts using the empirical prior estimate for the variance. Thus, DESeq2 differs from edgeR in estimating the width of the prior from the data, instead of using a user-adjustable parameter (R_0 in edgeR). Once DESeq2 has calculated the normalized read counts and the maximum *a posteriori* dispersal estimates for each sample it proceeds to calculating the log fold change values for each gene. Since small sample sizes may also lead to high heteroscedasticity of log fold changes, DESeq2 performs a second full empirical Bayes approach to shrink log fold change values. The empirical prior estimate for the log fold change is obtained by fitting a negative binomial distribution to the observed normalized counts, and dispersal estimates are shrunk by iteratively reweighing a least-squares algorithm. DESeq2 then obtains a prior density function for each gene's log fold change by fitting a zero-centered normal distribution to the empirical gene-wide maximum likelihood estimate distribution. The maximum *a posteriori* expression coefficient for each gene is given by the maximum value between the sum of the log-likelihoods for the negative binomial distribution and the log of the zero-centered normal distribution prior across all possible models. DESeq2 then uses all three estimates to obtain the overall expression strength and the log fold change between treatment and control for each gene. We used DESeq2's likelihood ratio test to compare expression strength between groups and test the hypothesis of differential gene expression. Prior to testing and to increase power, DESeq2 filters lowly expression genes by calculating the average expression strength of each gene, across all samples, omitting those whose normalized counts falls below a threshold which maximizes a user specified target false discovery rate. Both edgeR and DESeq2 account for multiple testing using the Benjamini and Hochberg procedure (Robinson *et al.*, 2010; Love *et al.*, 2014).

sleuth

Differential transcript expression need to take into account the fact that genes can be spliced into different isoforms. Dealing with read count variance in isoform expression is particularly challenging because as the number of isoform per gene increases, so does the uncertainty of expression estimates for any given isoform within that gene. sleuth (Pimentel *et al.*, 2017) uses both estimated transcript counts and estimated transcript count variance from kallisto to perform differential transcript expression analysis. Unlike DESeq2 and edgeR, sleuth uses an additive response error model (an extension of generalized linear models) instead of a negative binomial generalized linear model to model transcript abundances and partition its variance into a biological and a technical component. sleuth's model assumes that observed transcript abundances can be explained by a fixed effect inherent of each transcript and a 'biological noise' - assumed to be normally distributed. Due to the ambiguity of alignments, sleuth also incorporates uncertainty into transcript abundance estimation by assuming that the observed abundances for a given transcript can also be partitioned into the true read counts and an 'technical noise' - also assumed to be normally distributed. The technical variance is estimated as the mean of the sample variance obtained from the kallisto bootstraps, and the biological variance is estimated by subtracting the technical variance from the observed variance. sleuth starts by filtering low abundance transcripts (¡ 5 estimated counts in ¿= 47% of samples) from the dataset. Then, sleuth proceeds by performing two sequential normalization steps: one between, and one within samples. sleuth then uses DESeq2's Median-of-Ratios approach (Anders and Huber, 2010) to perform a between sample normalization, log transforming the weighted estimated counts to obtain a within sample normalization.

sleuth performs local variance shrinkage by splitting abundance values in 100 sliding windows across all transcripts, performing locally estimated scatterplot smoothing (LOESS) on a training set of transcripts in each window, and shrinking on the square root of the standard deviation (Law *et al.*, 2014). To test for differentially expressed transcripts between conditions sleuth applies a likelihood ration test between a model that includes sample labels (i.e. where samples are assigned to different conditions), and a model without labels (i.e. all samples are hypothesized to belong to the same condition). sleuth uses a false discovery rate control to obtain a list of differentially expressed genes and isoforms. To obtain a list of differentially expressed genes from sleuth we used sleuth's aggregate analysis of transcripts to obtain a final list of differentially expressed genes rather than transcripts. When comparing log fold change across methods we used sleuth's beta-value for transcripts to identify up or down regulated transcripts across comparisons.

Gene Set Enrichment Analyses

We performed gene set enrichment analyses to test the hypotheses that: Pigmented tissues up-regulate carotenoid and pteridine pathway genes relative to white tissues, while white tissues up-regulate guanine synthesis pathway genes relative to pigmented tissues; and yellow tissues up-regulate carotenoid pathway genes relative to orange tissues while orange tissues up-regulate pteridine pathway genes relative to yellow tissues. We implemented gene set enrichment analyses using three approaches: an over-representation test from R's package 'clusterProfiler' v.3.0.4 ('enrichKEGG'; Yu *et al.* 2020), and a self-contained enrichment test ('fry'; Wu *et al.* 2010) and a comparative enrichment test ('camera'; Wu and Smyth 2012) from R's package edgeR.

Over representation tests test the hypothesis that genes from a pre-determined gene set are disproportionately represented among differentially expressed genes. Prior to performing the over-representation test, we used KEGG's online blast KEGG Orthology And Links Annotation (blastKOALA; Kanehisa *et al.* 2016) to align query transcripts with open reading frames against the KEGG GENES database, and assigned a K number (a unique identifier of a functional ortholog within the KO database) to each transcript. We then used enrichKEGG to identify whether differentially expressed genes were disproportionately represented in one of the annotated KEGG pathways.

Self-contained enrichment tests test the hypothesis that genes in the gene set are differentially expressed between tissues. Fry performs a self-contained enrichment test by fitting a linear model to normalized expression values (calculated here as log transformed counts per million from edgeR)

and using an empirical Bayes t-statistic to test for gene-level differential expression (Wu *et al.*, 2010). Differently from typical enrichment tests, fry does not assume genes within a set to be independent. To account for correlation in expression between genes within a gene set, fry uses rotation, a MonteCarlo simulation for multivariate regression, instead of permutation during statistical testing.

Lastly, comparative enrichment tests test the hypothesis that genes in the gene set are more often differentially expressed (i.e. have higher rankings) than genes not in the gene set (i.e. in its complement). To compare rankings between tissue types, camera fits a linear model to normalized expression values using an empirical Bayes t-statistic to obtain a set of independent residuals for each gene in the test (Robinson *et al.*, 2010). These residuals are then standardized to have equal variance, summed over the genes, and then used to calculate the variance inflation factor expected to occur due to the correlation of genes belonging to the same pathway. Lastly, camera performs a t-test using the variance inflation factor to test the hypothesis that the average of the genewise statistic for the gene set of interest between tissue patches is equal to zero.

Supplementary Results

Genes Associated With Skin Permeability and Flexibility Are Differentially Expressed Between Belly and Dewlap Tissues

The lizard skin has two main functions: acting as barrier against external agents, and controlling the osmotic balance between the organism and its surrounding environment (Alibardi, 2003). A resistant and impermeable epidermis is obtained through the deposition of keratin and keratin-associated beta-proteins in the epidermis (Alibardi, 2003). Distinct regions of a lizard skin, however, have slightly different functions. On the one hand, the belly's main role is to protect an animal like *A. distichus*, a bark anole, against the abrasive action of tree barks; on the other hand, the dewlap's main role is communication, where the dewlap is repeatedly extended and retracted during displays. As a consequence, the arrangement of epidermal cells, the composition of the matrix surrounding these cells, and the genes and transcripts expressed by these cells varies throughout the skin (Rutland *et al.*, 2019). Therefore, in addition to looking for genes responsible for color and pattern when comparing white belly and orange and yellow pigmented samples, we were also interested in testing the hypothesis that white belly tissues would up-regulate genes that result in increased skin tenacity and reduced water loss relative to the pigmented dewlap skin.

As we expected, we found that belly tissues upregulated genes associated skin flexibility, resistance and permeability relative to dewlap tissues. Among differentially expressed genes between dewlap belly tissues we found an over-representation of genes with ontologies associated with the extracellular matrix (GO:0031012, GO:0005576, GO:0005615, GO:0030198), intermediate filament (GO:0005882), and cell adhesion (GO:0007155). Specifically, among these differentially expressed genes we identified genes responsible for the production of collagens, keratins and keratin associated beta-proteins.

We identified five differentially expressed collagen genes, four of which were upregulated in white belly tissues. In humans, these collagen genes play roles in the formation fibrils that make up connective tissues (*COL1A1*), regulate the formation of new fibrils (*COL14A1*), compose the basement membrane of subendothelium (*COL8A1*), and act as a cell-binding protein (*COL28A1*) (Bork, 1992). The one collagen gene upregulated in orange dewlap tissue forms components of the basal lamina of epithelial cells (*COL6A6*).

All but one keratins (reviewed in Bragulla and Homberger 2009) were upregulated in white belly tissues, including the most important of the keratin genes for basal keratinocytes: *KRT5* and *K14*. Consistent with a role of keratins in providing a higher resistance of belly relative to dewlap skin, *KRT10*, its paralog *KRT24*, and *KRT75* were also upregulated in white belly tissues. *KRT10* is a supra-basal epidermal keratin that increases the mechanical integrity of humans skin, while *KRT75* is responsible for the formation of harder epidermal structures in mammals, such as nails and hair. The only keratin upregulated in pigmented dewlaps was *KRT19*, which is typically expressed in simple epithelial cells and is hypothesized to have mostly regulatory rather than structural and mechanical functions.

We found two keratin associated beta-proteins (also known as beta-keratins) to be differentially expressed between dewlap and belly tissues: *LI-AC-30* is upregulated in the pigmented dewlap, while *LI-AC-27* is upregulated in the belly. Both of these genes produce glycine rich beta-proteins (Valle *et al.*, 2010). However, these proteins diverge in their glycine percentages: 30% of *LI-AC-27* and 8.2% of *LI-AC-30* are made up of glycines. Higher percentages of glycines are associated with more resistant tissues, which implies that the higher percentage of glycine in *LI-AC-27* likely results in a more resistant belly skin.

The identification of collagens, keratins and keratin-associated beta proteins highlights the multiple roles of the amniote skin. While dewlaps are extensible structures used inter and intra-specific displays, the belly is in constant contact with soil or bark, thus being an important barrier against the abrading effects of the substrate. It is also worth noting that keratins and beta-keratins have been found to play a role in avian coloration (Hill and McGraw, 2006). Therefore, we cannot rule out the possibility that these proteins play a role in determining color differences between the white belly and the pigment anole dewlaps.

Genes Responsible for Phenotypic Differences Between Parental Populations are Also Responsible For Differences Within Bi-colored Specimens

Even though our main aim was to use differential expression to test identify candidate genes for color and color pattern in squamates, we were also interested in testing the hypothesis of whether the same molecular mechanisms responsible for phenotypic differences within a specimen are also responsible for differences between specimens. To do so we performed differential expression analyses for each tissue comparison using a paired and an unpaired experimental design. A paired design compares pairs of observations from biological replicates, with each observation belonging to one of the two variables of interest (e.g. using both samples from a specimen with bi-colored dewlaps). Thus, paired designs control for specimen identity when fitting the data to a generalized linear model. An unpaired design, on the other hand, uses from all different specimens - not controlling for specimen identification. Specifically, for each tissue comparison we assessed whether expression profiles were consistent across experimental designs. We predicted that if the same mechanisms are responsible for phenotypic differences within and between specimens, we

would: (a) find large number of differentially expressed genes shared between paired and unpaired designs, and (b) find a strong positive correlation in log-fold change estimates across genes between tissues.

By comparing the sets of differentially expressed genes and calculating the correlation of log-fold change estimates between paired and unpaired experimental designs, we confirm our prediction that same molecular mechanisms are likely responsible for differences in tissue colors both between populations and within specimens with bi-colored dewlaps. However, even though correlation slopes varied between 0.35 and 0.64 (p ¡ 0.05 across all comparisons), there was only partial overlap of differentially expressed genes in comparisons that included orange tissues. Upon further inspection, we found that most differentially expressed genes from paired designs that included orange gular tissues consisted of muscle proteins. The enrichment of muscle proteins in gular tissues is likely due to the positioning of dewlap muscles at the basis of jaw, but not extending into the dewlap margins (Font and Rome, 1990). Despite the over-representation of muscle proteins in comparisons with orange gular tissues, log-fold change across experimental designs within tissue comparisons as consistent (i.e. genes were up-regulated in the same tissue across experimental designs) for 514 out of the 548 candidate genes. The positive and consistent correlation of log-fold change across all experimental designs suggest that despite the noise due to muscle tissue in the dewlap gular dermis in paired designs, there is a consistent signal in the data associated with differences in skin phenotype across designs. Therefore we conclude that this data corroborates our prediction that the same mechanisms responsible for differences in phenotype between specimens are also likely responsible for differences in phenotypes within a specimen with a bi-colored dewlap.

References

Alibardi, L. 2003. Adaptation to the land: the skin of reptiles in comparison to that of amphibians and endotherm amniotes. *Journal of Experimental Zoology Part B: Molecular and Developmental Evolution*, 298(1): 12–41.

Anders, S. and Huber, W. 2010. Differential expression analysis for sequence count data. *Nature Precedings*, pages 1–3.

Bork, P. 1992. The modular architecture of vertebrate collagens. *FEBS Letters*, 307(1): 49–54.

Bragulla, H. H. and Homberger, D. G. 2009. Structure and functions of keratin proteins in simple, stratified, keratinized and cornified epithelia. *Journal of Anatomy*, 214(4): 516–559.

Bray, N. L., Pimentel, H., Melsted, P., and Pachter, L. 2016. Near-optimal probabilistic RNA-seq quantification. *Nature Biotechnology*, 34(5): 525–527.

Deschamps-Francoeur, G., Simoneau, J., and Scott, M. S. 2020. Handling multi-mapped reads in RNA-seq. *Computational and Structural Biotechnology Journal*, 18: 1569–1576.

Font, E. and Rome, L. C. 1990. Functional morphology of dewlap extension in the lizard *Anolis equestris* (Iguanidae). *Journal of Morphology*, 206(2): 245–258.

Hill, G. E. and McGraw, K. J. 2006. *Bird Coloration: Mechanisms and measurements*. Harvard University Press, Cambridge, MA.

Kanehisa, M., Sato, Y., and Morishima, K. 2016. BlastKOALA and GhostKOALA: KEGG Tools for Functional Characterization of Genome and Metagenome Sequences. *Journal of Molecular Biology*, 428(4): 726–731.

Langmead, B. and Salzberg, S. L. 2012. Fast gapped-read alignment with Bowtie 2. *Nature Methods*, 9(4): 357–359.

Law, C. W., Chen, Y., Shi, W., and Smyth, G. K. 2014. voom: precision weights unlock linear model analysis tools for RNA-seq read counts. *Genome Biology*, 15(2): 1–17.

Li, B. and Dewey, C. N. 2011. RSEM: accurate transcript quantification from RNA-Seq data with or without a reference genome. *BMC Bioinformatics*, 12(1): 1–16.

Love, M. I., Huber, W., and Anders, S. 2014. Moderated estimation of fold change and dispersion for RNA-seq data with DESeq2. *Genome Biology*, 15(12): 1–21.

Patro, R., Duggal, G., Love, M. I., Irizarry, R. A., and Kingsford, C. 2017. Salmon provides fast and bias-aware quantification of transcript expression. *Nature Methods*, 14(4): 417–419.

Pimentel, H., Bray, N. L., Puente, S., Melsted, P., and Pachter, L. 2017. Differential analysis of rna-seq incorporating quantification uncertainty. *Nature methods*, 14(7): 687.

Robinson, M. D. and Oshlack, A. 2010. A scaling normalization method for differential expression analysis of RNA-seq data. *Genome Biology*, 11(3): 139–140.

Robinson, M. D., McCarthy, D. J., and Smyth, G. K. 2010. edgeR: a Bioconductor package for differential expression analysis of digital gene expression data. *Bioinformatics (Oxford, England)*, 26(1): 139–140.

Rutland, C. S., Cigler, P., and Kubale, V. 2019. Reptilian skin and its special histological structures. In C. S. Rutland and V. Kubale, editors, *Veterinary Anatomy and Physiology*, chapter 8. IntechOpen, Rijeka.

Srivastava, A., Sarkar, H., Gupta, N., and Patro, R. 2016. RapMap: a rapid, sensitive and accurate tool for mapping RNA-seq reads to transcriptomes. *Bioinformatics*, 32(12): i192–i200.

Valle, L. D., Nardi, A., Bonazza, G., Zuccal, C., Emera, D., and Alibardi, L. 2010. Forty keratin-associated -proteins (-keratins) form the hard layers of scales, claws, and adhesive pads in the green anole lizard, Anolis carolinensis. *Journal of Experimental Zoology Part B: Molecular and Developmental Evolution*, 314B(1): 11–32.

Wu, D. and Smyth, G. K. 2012. Camera: a competitive gene set test accounting for inter-gene correlation. *Nucleic Acids Research*, 40(17): 1–12.

Wu, D., Lim, E., Vaillant, F., Asselin-Labat, M.-L., Visvader, J. E., and Smyth, G. K. 2010. ROAST: rotation gene set tests for complex microarray experiments. *Bioinformatics*, 26(17): 2176–2182.

Yu, G., Wang, L.-G., and Dall'Olio, G. 2020. clusterProfiler: statistical analysis and visualization of functional profiles for genes and gene clusters.

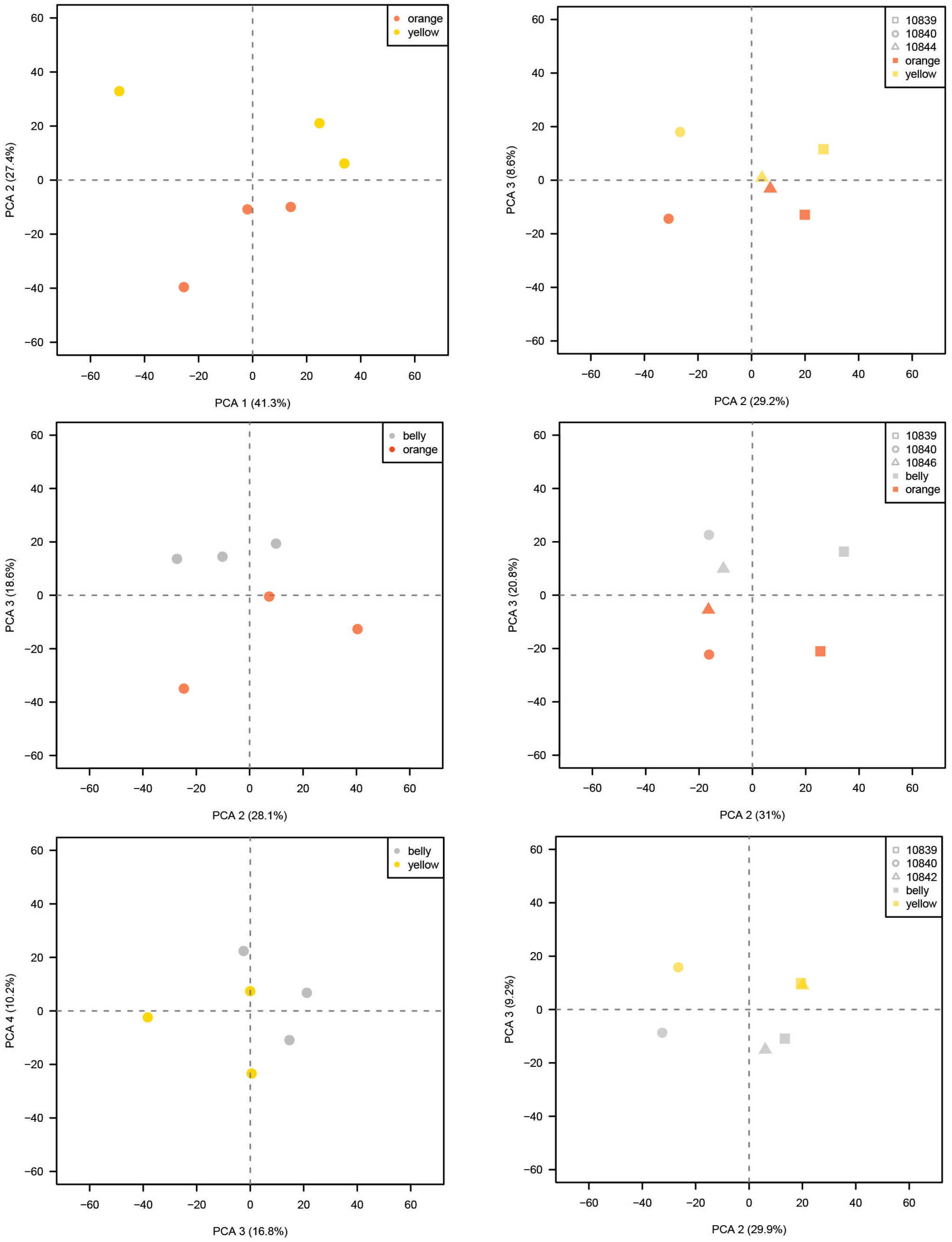

Fig. S1. Principal component analysis plot of normalized expected read counts for both pairwise comparisons and experimental designs. Right column: unpaired experimental design; left column: paired experimental design. Top row: orange vs. yellow skin comparison; center row: white belly vs. orange skin comparison; bottom row: white belly vs. yellow skin comparison. In paired designs, identical shapes represent samples with different colors obtained from the same specimen. Notice how for all comparisons one can visualize clusters according to tissue colors, and how differences between yellow and white tissues occur in principal components that explain a smaller amount of variation than comparisons between orange and white tissues.

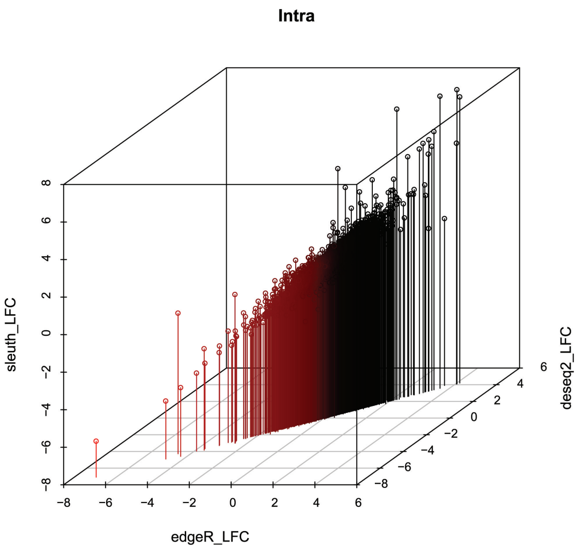

Fig. S2. Correlation between log-fold change estimates per gene across three differential expression pipelines for the comparison between white and pigmented tissues under the paired experimental design.

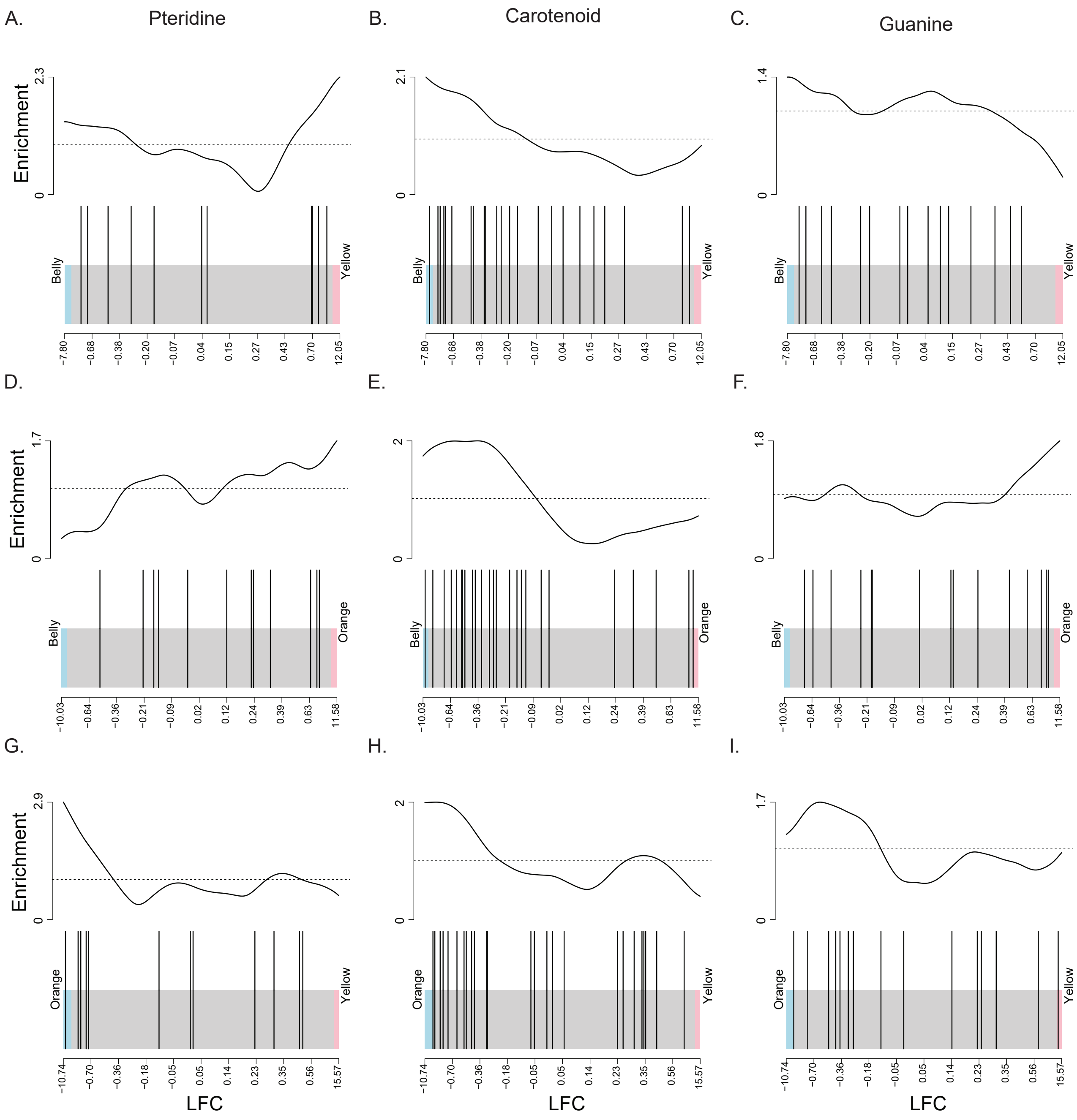

Fig. S3. Barcode plot comparing the pteridine (A,D,G), carotenoid (B,E,H), and guanine (C,F,I) pathways across unpaired experimental designs between (A-C) white belly and yellow dewlap skin, (D-F) white belly and orange dewlap skin, and (G-I) orange and yellow dewlap skin. Genes are ranked by increasing log-fold change from left to right, and the genes in each pathway are represented by vertical bars. The curve above the barcode represents the local enrichment for genes in the pathway. Mixed enrichment tests mimic the patterns observed in these barcode plots, supporting the prediction that distinct parts of these pathways were upregulated in skin samples with different colors. These results are particularly noticeable in the barcode plots for the carotenoid pathway between orange and white skin, and in the pteridine pathway between orange and yellow skin.

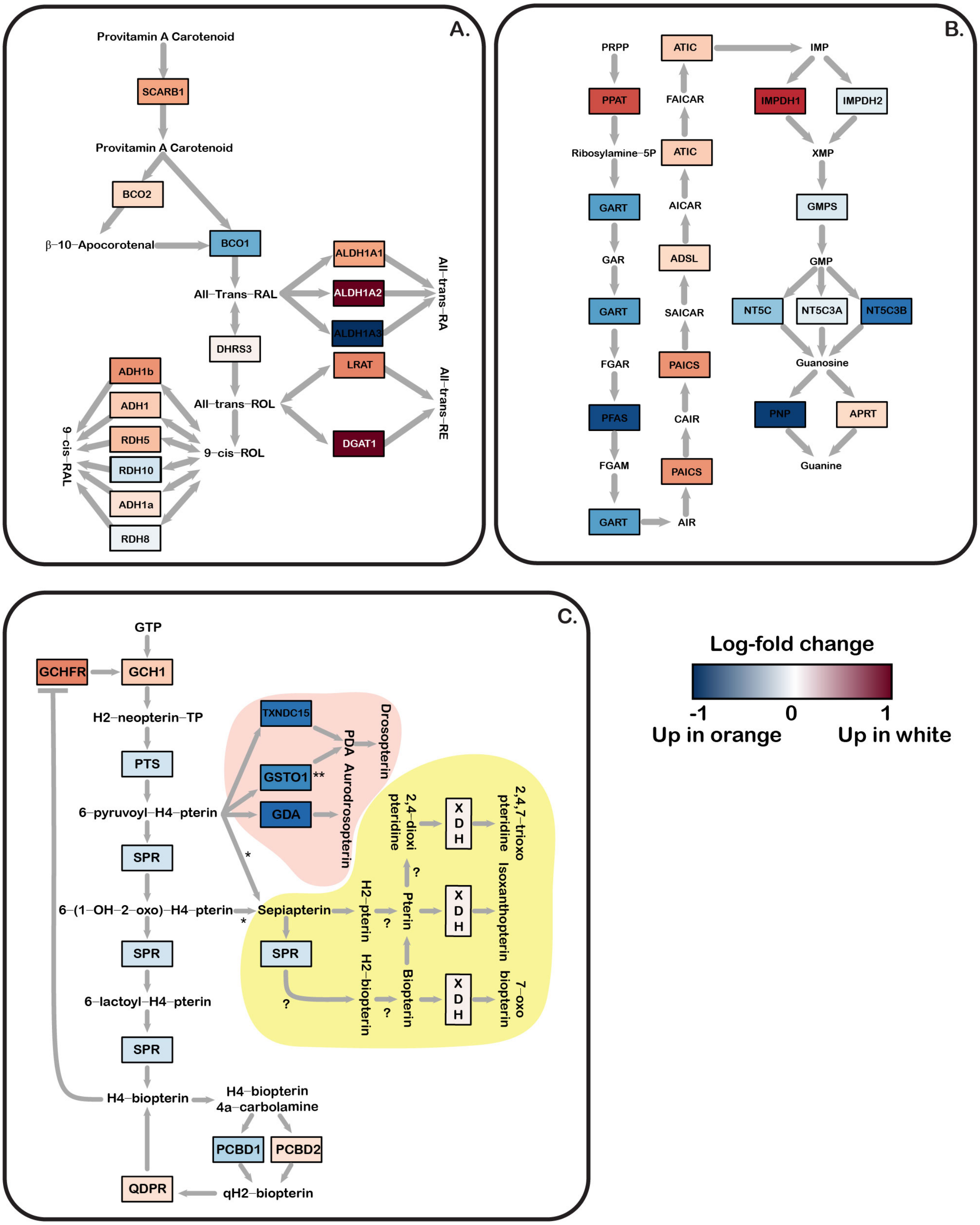

Fig. S4. Illustration of log-fold changes from comparisons between white and orange samples for unpaired designs across the carotenoid (A), guanine (B) and pteridine (C) pathways. '*' represent hypothetical reactions; '**' represent candidate based on similarity to candidate drosophilid gene; '?' represent unidentified enzymes. Pink blob represents set of reactions in pteridine pathway responsible for the production of red/orange pigments; yellow blob represents set of reactions in pteridine pathway responsible for the production of yellow pigments.

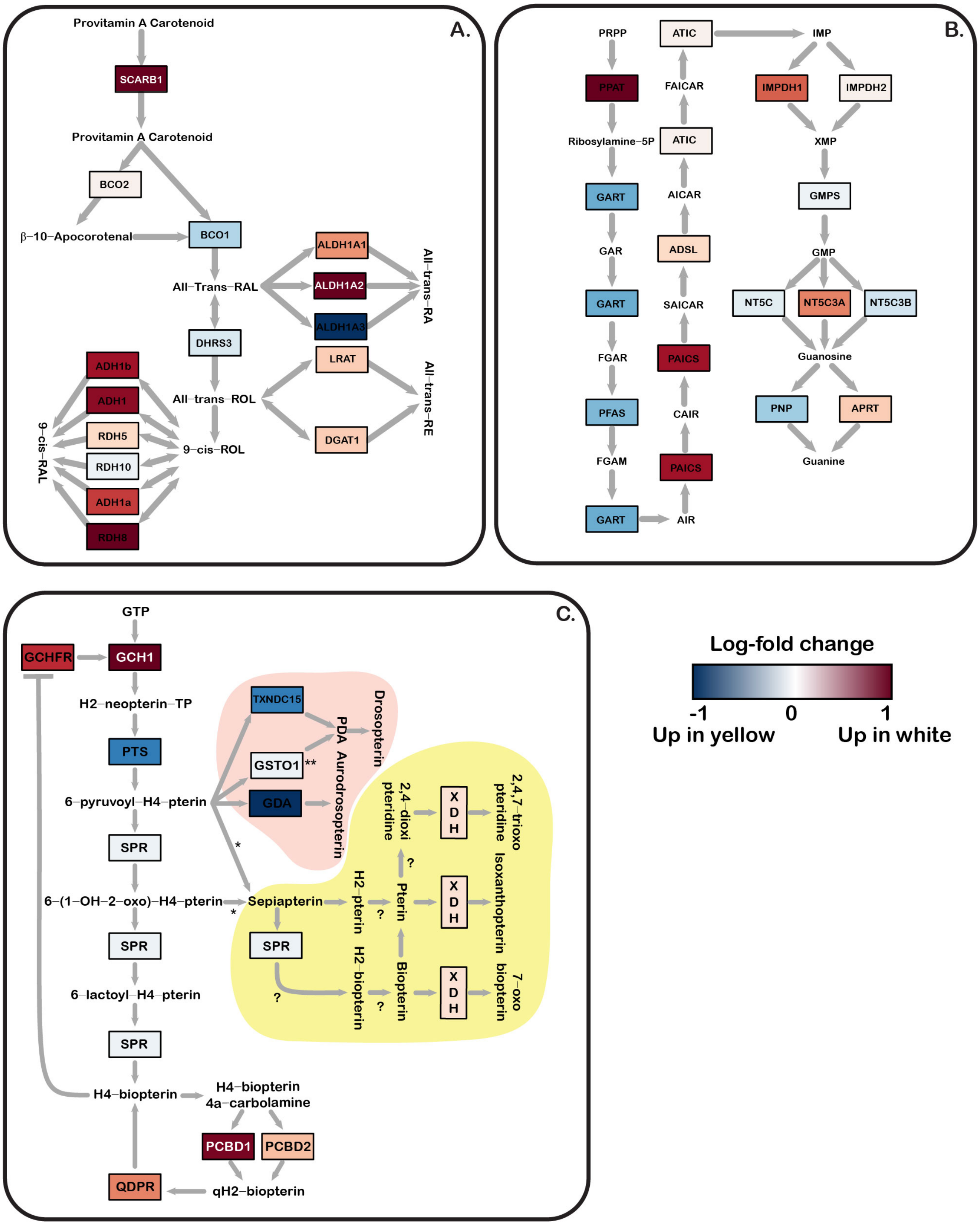

Fig. S5. Illustration of log-fold changes from comparisons between white and yellow samples for unpaired designs across the carotenoid (A), guanine (B) and pteridine (C) pathways. '*' represent hypothetical reactions; '**' represent candidate based on similarity to candidate drosophilid gene; '?' represent unidentified enzymes. Pink blob represents set of reactions in pteridine pathway responsible for the production of red/orange pigments; yellow blob represents set of reactions in pteridine pathway responsible for the production of yellow pigments.

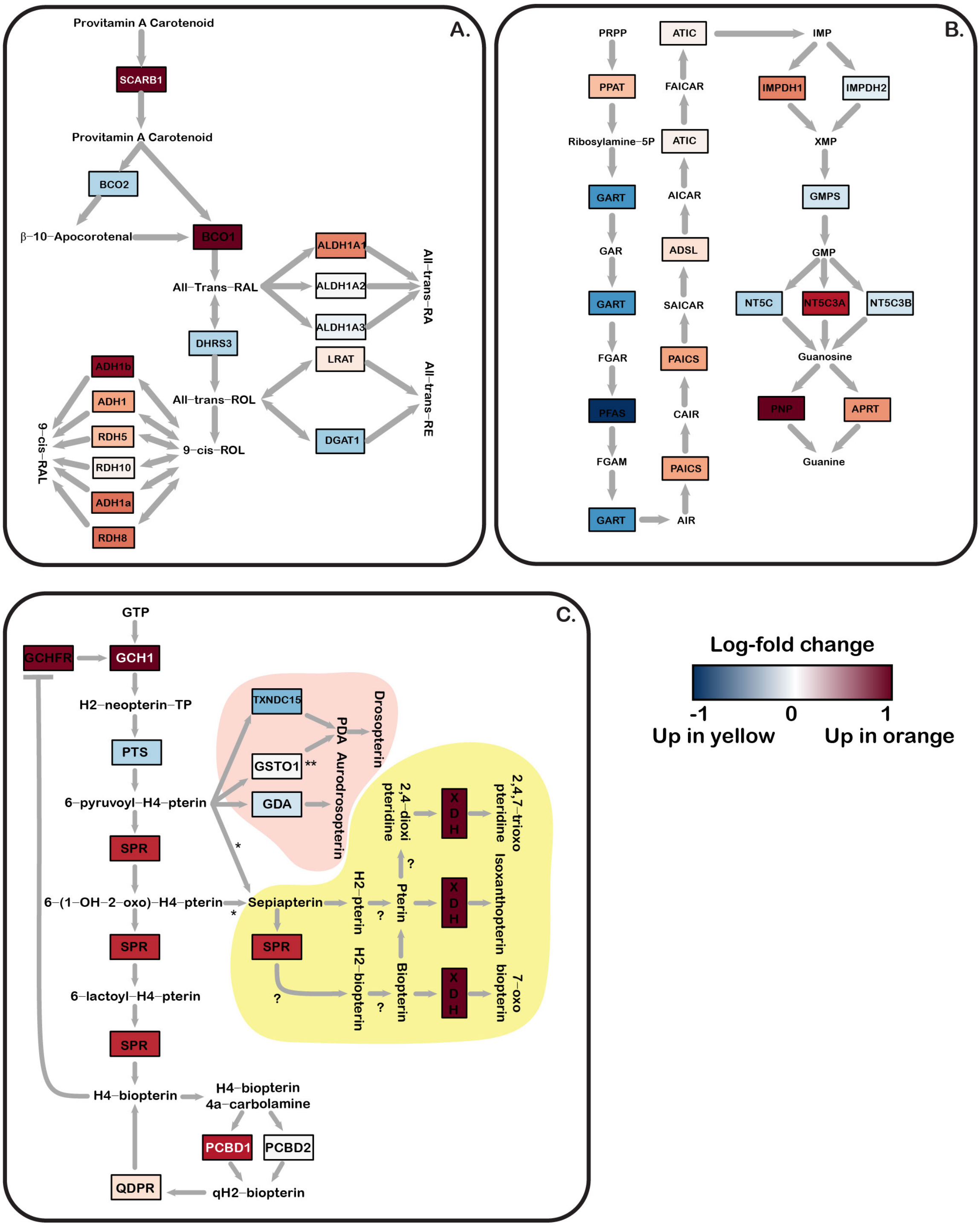

Fig. S6. Illustration of log-fold changes from comparisons between orange and yellow samples for unpaired designs across the carotenoid (A), guanine (B) and pteridine (C) pathways. '*' represent hypothetical reactions; '**' represent candidate based on similarity to candidate drosophilid gene; '?' represent unidentified enzymes. Pink blob represents set of reactions in pteridine pathway responsible for the production of red/orange pigments; yellow blob represents set of reactions in pteridine pathway responsible for the production of yellow pigments.

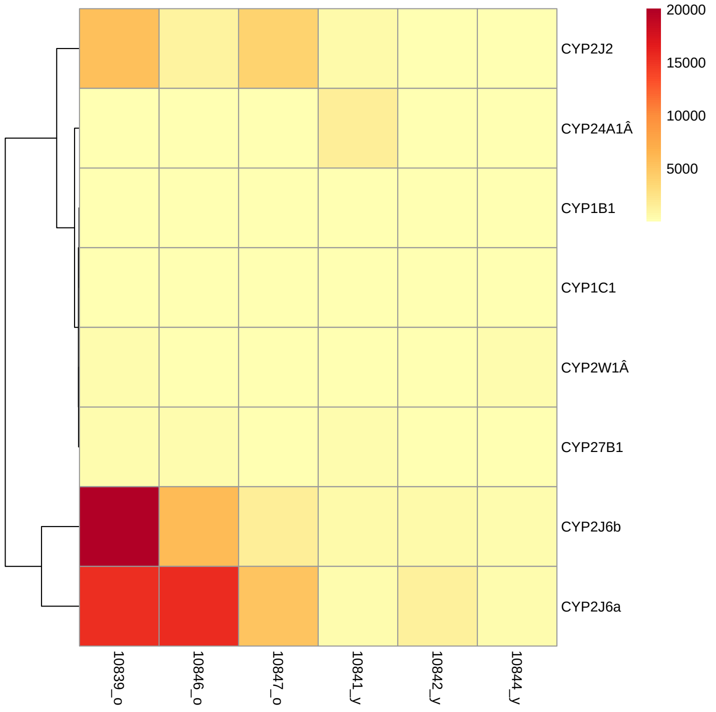

Fig. S7. Heatmap of normalized counts under an unpaired expression design for differentially expressed Cytochrome P450 genes. The two transcripts annotated to *CYP2J6* show a larger expression in orange dewlaps than in yellow dewlaps.

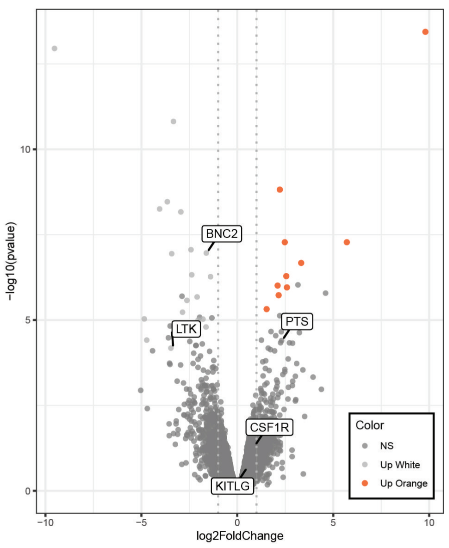

Fig. S8. Scatter-plots between $\log_2$-fold change and $-\log_{10}$ transformed p-values (i.e. volcano plots) for unpaired comparisons between orange and belly samples. Each dot represents a gene, and the dotted light grey line represents $\log_2$-fold changes of -1 and 1. We assigned colors to dots depending on their significance or lack thereof. We considered samples with a false-discovery rate ≤ 0.05 for the Fisher's combined test across experimental designs and differential expression pipelines as significant. Orange dots - significantly up-regulated in orange tissues; light grey dots - significantly up-regulated in white tissues. We labeled candidate genes in the volcano plots for visualization.

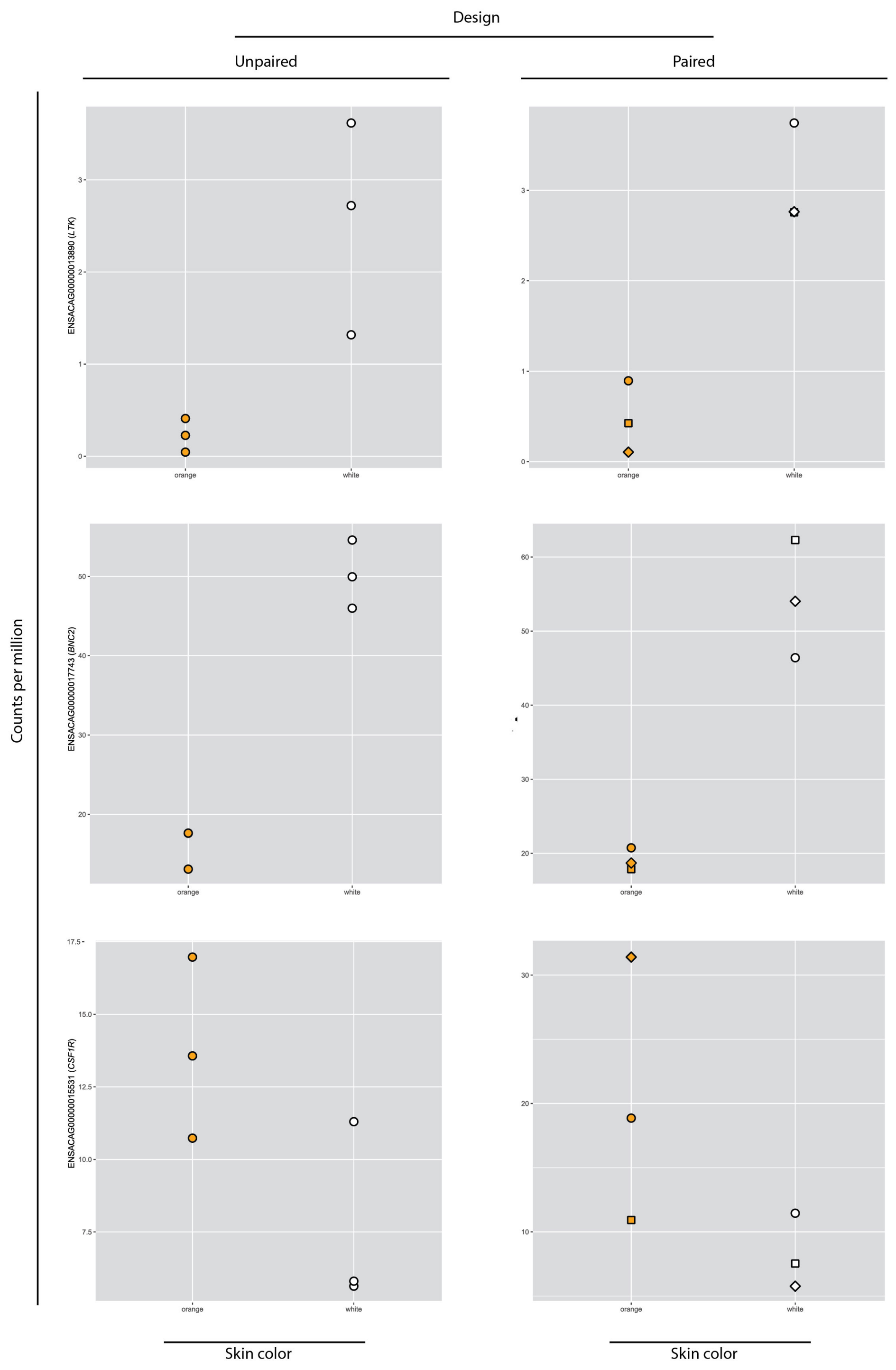

Fig. S9. Visual depiction of differences in counts per million (CPM) reads for candidate genes between skin colors across paired and unpaired designs. Each column represents a single gene. We provide both the ENSEMBL identification number and common gene abbreviation for each gene. In paired and unpaired designs, distinct colors represent distinct skin colors, while in paired designs geometrical shapes represent skin colors sampled from the same specimen. In paired designs, notice the difference in CPM within specimens. This plot represents differences in CPM between orange and white skin for candidate genes associated with color pattern.

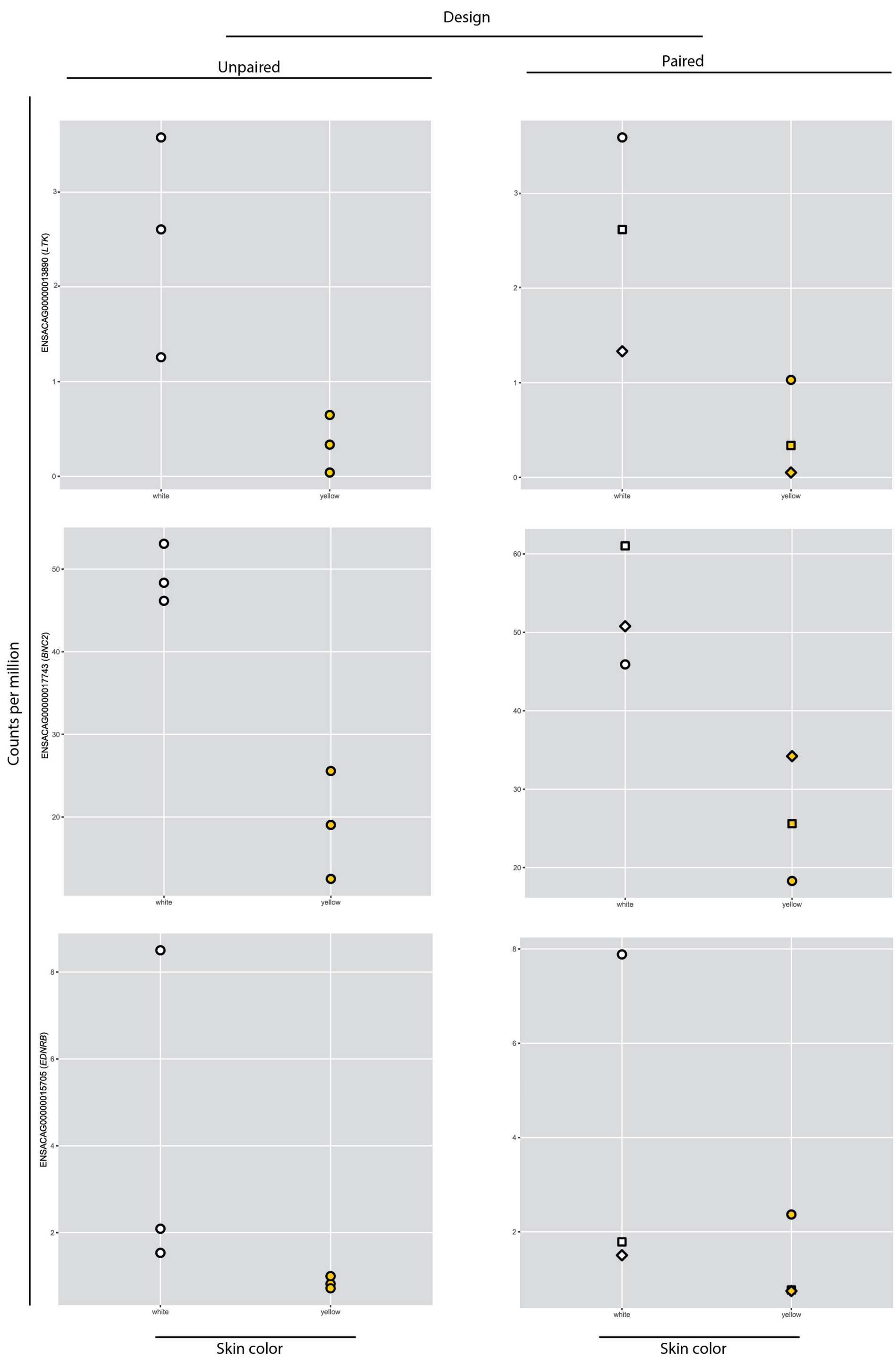

Fig. S10. Visual depiction of differences in counts per million (CPM) reads for candidate genes between skin colors across paired and unpaired designs. Each column represents a single gene. We provide both the ENSEMBL identification number and common gene abbreviation for each gene. In paired and unpaired designs, distinct colors represent distinct skin colors, while in paired designs geometrical shapes represent skin colors sampled from the same specimen. In paired designs, notice the difference in CPM within specimens. This plot represents differences in CPM between yellow and white skin for candidate genes associated with color pattern.

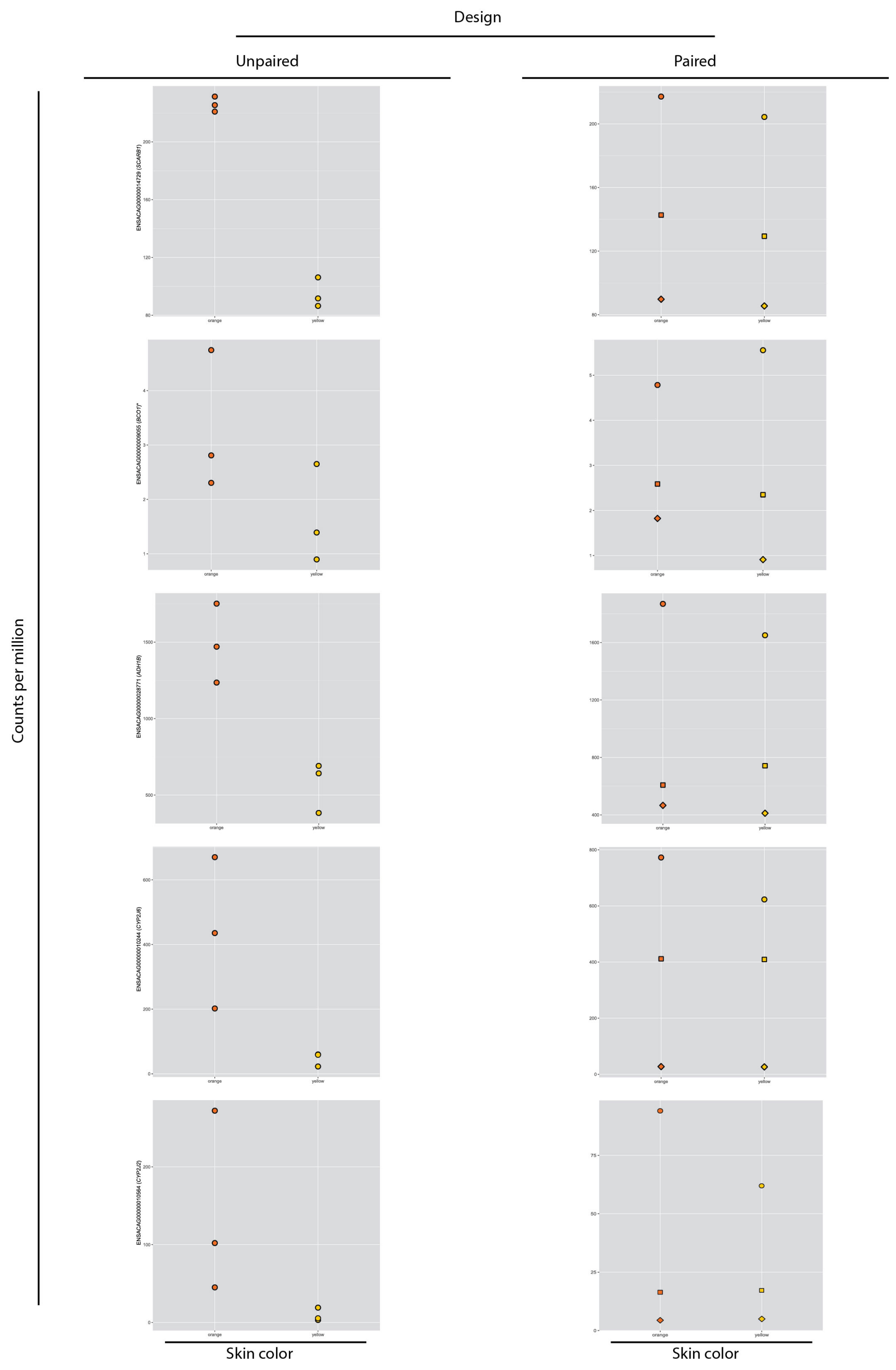

Fig. S11. Visual depiction of differences in counts per million (CPM) reads for candidate genes between skin colors across paired and unpaired designs. Each column represents a single gene. We provide both the ENSEMBL identification number and common gene abbreviation for each gene. In paired and unpaired designs, distinct colors represent distinct skin colors, while in paired designs geometrical shapes represent skin colors sampled from the same specimen. In paired designs, notice the difference in CPM within specimens. This plot represents differences in CPM between orange and yellow skin for candidate genes associated with color.

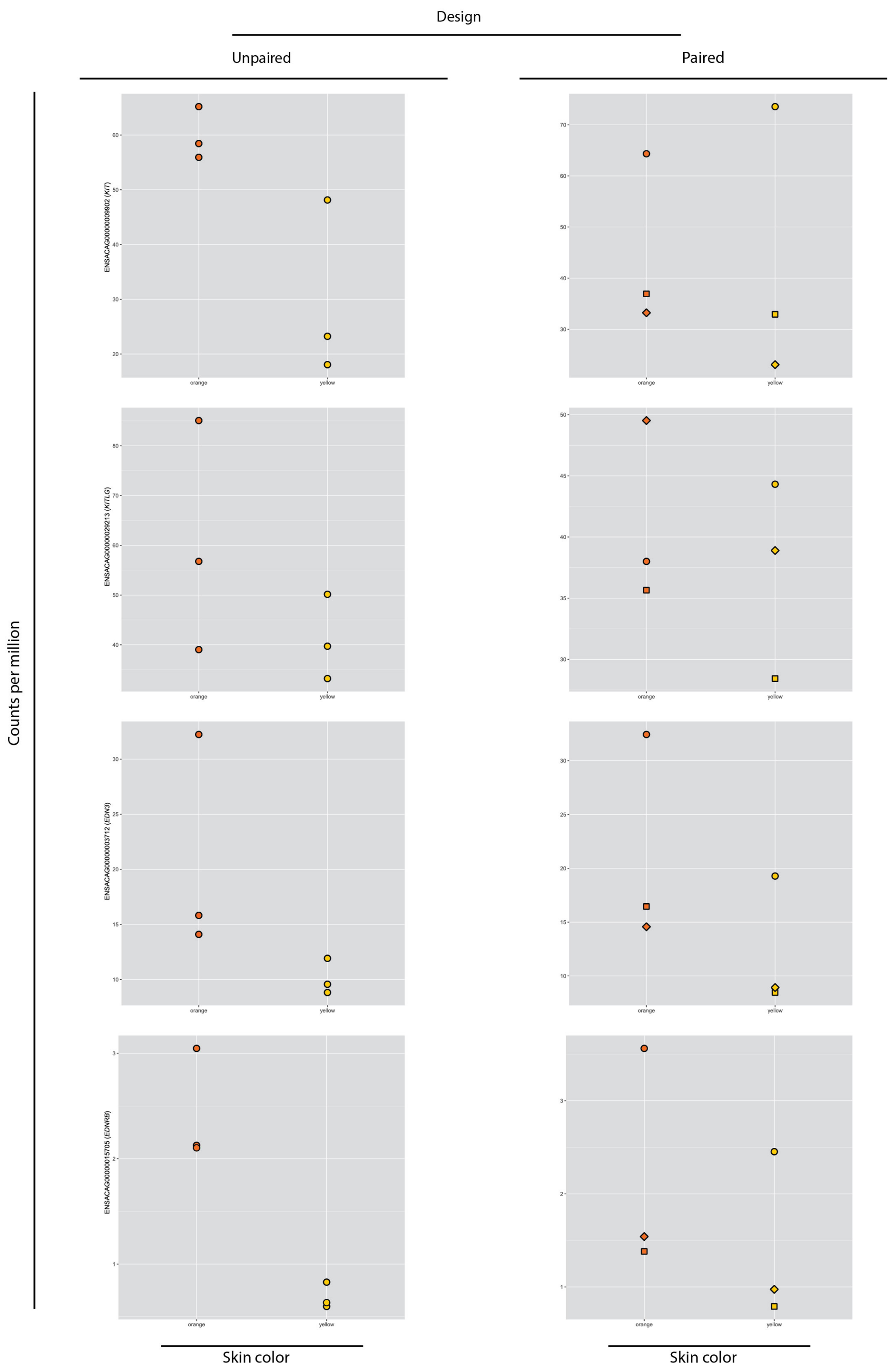

Fig. S12. Visual depiction of differences in counts per million (CPM) reads for candidate genes between skin colors across paired and unpaired designs. Each column represents a single gene. We provide both the ENSEMBL identification number and common gene abbreviation for each gene. In paired and unpaired designs, distinct colors represent distinct skin colors, while in paired designs geometrical shapes represent skin colors sampled from the same specimen. In paired designs, notice the difference in CPM within specimens. This plot represents differences in CPM between orange and yellow skin for candidate genes associated with color pattern.
